# Functional *IL6R* 358Ala Allele Impairs Classical IL-6 Receptor Signaling and Influences Risk of Diverse Inflammatory Diseases

**DOI:** 10.1371/journal.pgen.1003444

**Published:** 2013-04-04

**Authors:** Ricardo C. Ferreira, Daniel F. Freitag, Antony J. Cutler, Joanna M. M. Howson, Daniel B. Rainbow, Deborah J. Smyth, Stephen Kaptoge, Pamela Clarke, Charlotte Boreham, Richard M. Coulson, Marcin L. Pekalski, Wei-Min Chen, Suna Onengut-Gumuscu, Stephen S. Rich, Adam S. Butterworth, Anders Malarstig, John Danesh, John A. Todd

**Affiliations:** 1 Juvenile Diabetes Research Foundation/Wellcome Trust Diabetes and Inflammation Laboratory, Department of Medical Genetics, NIHR Cambridge Biomedical Research Centre, Cambridge Institute for Medical Research, University of Cambridge, Cambridge, United Kingdom; 2 Department of Public Health and Primary Care, Strangeways Research Laboratory, University of Cambridge, Cambridge, United Kingdom; 3 Center for Public Health Genomics, University of Virginia, Charlottesville, Virginia, United States of America; 4 Precision Medicine, Pfizer Global Research and Development, Cambridge, United Kingdom; Georgia Institute of Technology, United States of America

## Abstract

Inflammation, which is directly regulated by interleukin-6 (IL-6) signaling, is implicated in the etiology of several chronic diseases. Although a common, non-synonymous variant in the IL-6 receptor gene (*IL6R* Asp358Ala; rs2228145 A>C) is associated with the risk of several common diseases, with the 358Ala allele conferring protection from coronary heart disease (CHD), rheumatoid arthritis (RA), atrial fibrillation (AF), abdominal aortic aneurysm (AAA), and increased susceptibility to asthma, the variant's effect on IL-6 signaling is not known. Here we provide evidence for the association of this non-synonymous variant with the risk of type 1 diabetes (T1D) in two independent populations and confirm that rs2228145 is the major determinant of the concentration of circulating soluble IL-6R (sIL-6R) levels (34.6% increase in sIL-6R per copy of the minor allele 358Ala; rs2228145 [C]). To further investigate the molecular mechanism of this variant, we analyzed expression of IL-6R in peripheral blood mononuclear cells (PBMCs) in 128 volunteers from the Cambridge BioResource. We demonstrate that, although 358Ala increases transcription of the soluble *IL6R* isoform (*P* = 8.3×10^−22^) and not the membrane-bound isoform, 358Ala reduces surface expression of IL-6R on CD4+ T cells and monocytes (up to 28% reduction per allele; *P*≤5.6×10^−22^). Importantly, reduced expression of membrane-bound IL-6R resulted in impaired IL-6 responsiveness, as measured by decreased phosphorylation of the transcription factors STAT3 and STAT1 following stimulation with IL-6 (*P*≤5.2×10^−7^). Our findings elucidate the regulation of IL-6 signaling by IL-6R, which is causally relevant to several complex diseases, identify mechanisms for new approaches to target the IL-6/IL-6R axis, and anticipate differences in treatment response to IL-6 therapies based on this common *IL6R* variant.

## Author Summary

Interleukin-6 (IL-6) is a complex cytokine, which plays a critical role in the regulation of inflammatory responses. Genetic variation in the IL-6 receptor gene is associated with the risk of several human diseases with an inflammatory component, including coronary heart disease, rheumatoid arthritis, and asthma. A common non-synonymous single nucleotide polymorphism in this gene (Asp358Ala) has been suggested to be the causal variant in this region by affecting the circulatory concentrations of soluble IL-6R (sIL-6R). In this study we extend the genetic association of this variant to type 1 diabetes and provide evidence that this variant exerts its functional mechanism by regulating the balance between sIL-6R (generated through cleavage of the surface receptor and by alternative splicing of a soluble *IL6R* isoform) and membrane-bound IL-6R. These data show for the first time that the minor allele of this non-synonymous variant (Ala358) directly controls the surface levels of IL-6R on individual immune cells and that these differences in protein levels translate into a functional impairment in IL-6R signaling. These findings may have implications for clinical trials targeting inflammatory mechanisms involving IL-6R signaling and may provide tools for identifying patients with specific benefit from therapeutic intervention in the IL-6R signaling pathway.

## Introduction

Originally identified as a B-cell differentiation factor, interleukin-6 (IL-6) is now recognized as one of the most pleiotropic cytokines in humans. IL-6 can activate a wide-range of cell types and is recognized as a critical regulator of acute inflammatory reactions [1]. In addition, IL-6 plays a key role in controlling the activation and differentiation of T-cell responses, promoting a pro-inflammatory environment, which has been associated with the pathogenesis of several autoimmune and inflammatory diseases in humans [2].

Binding of IL-6 to the membrane-bound IL-6 receptor (IL-6R) induces homodimerization with its co-receptor gp130, resulting in the phosphorylation of the transcription factors STAT3 and STAT1 (classical signaling) [3]. Alternatively, a circulating soluble form of IL-6R (sIL-6R), if bound to IL-6, is able to stimulate cells expressing gp130 (“trans-signaling”), even in the absence of membrane-bound IL-6R [4]. The role of genetic variation in *IL6R* in the etiology of human disease has been highlighted by genetic studies reporting the association of variants in the gene with the risk of several diseases with an inflammatory component, including coronary heart disease (CHD) [5]–[7], rheumatoid arthritis (RA) [8], atrial fibrillation (AF) [9], abdominal aortic aneurysm (AAA) [10] and asthma [11]. A common (MAF 30–40% in European and Asian HapMap populations) non-synonymous variant Asp358Ala in *IL6R* (rs2228145 A>C, previously rs8192284) has been suggested to be the causal variant at this locus, because of its strong correlation with circulating concentrations of sIL-6R [12]. However, the effect of this variant on classical IL-6R signaling remains unclear. Here we demonstrate that the 358Ala allele regulates IL-6R surface expression at the protein level in specific immune cell subsets, resulting in altered IL-6 signaling. These findings clearly demonstrate the effect of Asp358Ala in the regulation of classical IL-6 signaling and provide further insight into the functional mechanism underpinning the association of this genetic variant with human diseases.

## Results

Given the association of rs2228145 with a variety of human inflammatory diseases, we assessed its association with type 1 diabetes (T1D). We found evidence for a protective effect of 358Ala in 8,371 T1D patients and 10,092 unrelated healthy controls (*P* = 0.0092, OR [95% CI] = 0.94 [0.91–0.99]; Figure S1). We replicated these findings in an independent collection of 3,771 T1D families (*P* = 0.0035, OR [95% CI] = 0.92 [0.87–0.97]; Figure S1). These data implicate the *IL6R* locus in the etiology of T1D, with a consistent protective effect as reported for CHD, RA, AF and AAA (Figure S1).

**Figure 1:**
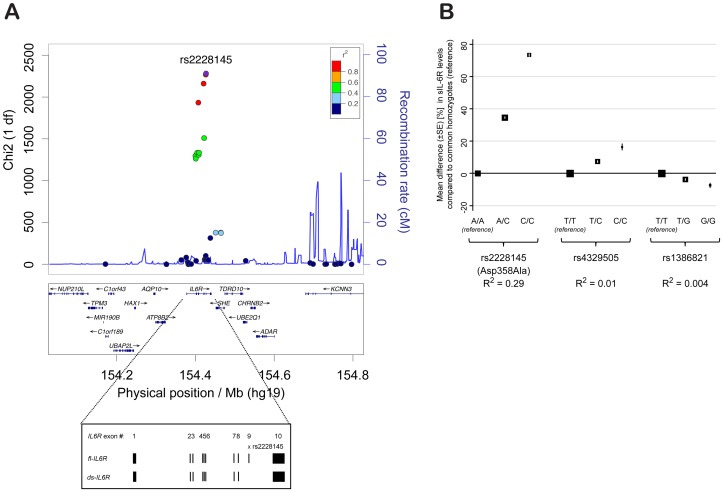
rs2228145 is the major determinant of circulating sIL-6R levels at the *IL6R* locus. (A) X^2^ statistics for the association (additive allelic effects model of inheritance; see Methods) of 45 SNPs genotyped using the Illumina ImmunoChip with sIL-6R concentrations are plotted against the physical position of the SNPs on chromosome 1 (hg.19). Recombination rates and linkage disequilibrium (r^2^) values are based on CEU HapMap. Inset depicts exonic structure of the membrane-bound IL-6R (*fl-IL6R*) and the differentially spliced soluble isoform (*ds-IL6R*). (B) Results from a regression model containing all three independent IL6R SNPs. Mean differences in sIL-6R concentration (%) compared to the common homozygote reference groups are plotted for the 3 SNPs independently associated with sIL-6R levels. Box size is proportional to the number of individuals in each group and error bars represent the standard error of the mean. SE = standard error of the mean difference. R^2^ = variance explained by the individual SNP.

The minor allele of rs2228145 (358Ala) has been shown to be strongly associated with increased concentrations of circulating sIL-6R [5], [12] and has, therefore, been assumed to be the causal allele in the *IL6R* locus. To confirm that rs2228145 is the major determinant of circulating sIL-6R levels in *IL6R*, and to identify potential additional genetic determinants of sIL-6R levels at this locus, we correlated circulating sIL-6R concentrations measured in 3,605 individuals with 45 SNPs genotyped at the *IL6R* locus using the Illumina ImmunoChip [13] (Figure 1A and Table S1). Twenty four SNPs were associated with sIL-6R at *P*<5×10^−8^, including rs2228145, which had the strongest evidence of association (chi2(2df) = 2296, *P*<10^−300^). These differences equated to an increase of approximately 35% in the concentrations of sIL-6R for each copy of the 358Ala allele (Figure 1B and Table S2). Using a forward stepwise regression analysis, two additional intronic SNPs, rs4329505 (r^2^ = 0.113; D′ = 1 with rs2228145 in CEU) and rs1386821 (r^2^ = 0.001; D′ = 0.064 with rs2228145 in CEU) were both found to be independently associated with circulating sIL-6R (*P* = 7.4×10^−29^ and *P* = 5.1×10^−11^). However, their effects were substantially smaller (R^2^ = 1.1% and 0.4%, respectively) compared to R^2^ = 29.3% for rs2228145 (Figure 1B and Table S2).

Previous studies support two independent processes for the generation of circulating sIL-6R: i) transcriptional regulation of a differentially spliced isoform (*ds-IL6R*; Figure 1A) [14] and ii) increased proteolytic cleavage of the membrane-bound receptor [15], [16]. While it has been speculated that 358Ala affects circulating sIL-6R through both of these processes, there is no comprehensive evidence on the mechanism of the variant, especially with regards to surface IL-6R expression and signaling.

**Figure 2:**
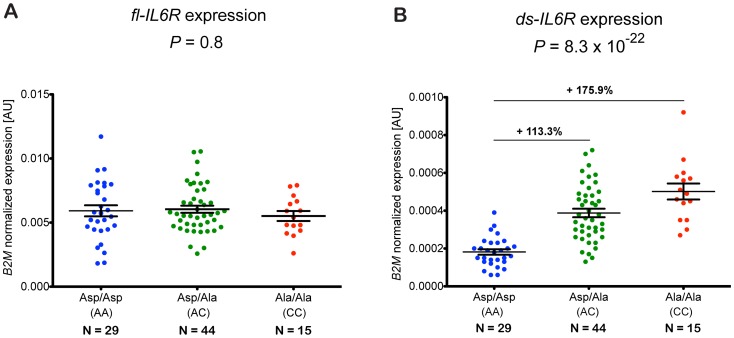
rs2228145 regulates the expression of the *ds-IL6R* but not the *fl-IL6R* isoform. (A) Expression of *fl-IL6R* and (B) *ds-IL6R* relative to the housekeeping gene *B2M* was measured by quantitative real-time PCR of RNA purified from PBMCs of 88 healthy volunteers from the Cambridge BioResource. Individual expression levels and their mean (±SEM) are plotted according to rs2228145 genotype. Differences in the mean expression levels relative to the common homozygotes group (Asp/Asp) are indicated above the black horizontal lines. *P*-values represent test for an association of rs2228145 with the expression levels of *fl-IL6R* or *ds-IL6R*, using an additive allelic effects model. AU, arbitrary units.

To investigate the effect of rs2228145 on the transcriptional regulation of *IL6R* in human primary cells, we designed a qPCR assay to measure the relative expression levels of both the full length *IL6R* isoform (*fl-IL6R*), encoding the membrane-bound receptor, and the *ds-IL6R* isoform, which lacks exon 9 containing the IL-6R trans-membrane domain, and hence encodes sIL-6R. Importantly, in PBMCs from 88 healthy CBR donors (Table S3), the expression of *fl-IL6R* was not significantly different according to rs2228145 genotype (*P* = 0.8, Figure 2A). Consistent with a previous report on multiple myeloma plasma cells [17], there was a significant increase of *ds-IL6R* expression in carriers of the 358Ala allele (*P* = 8.3×10^−22^, Figure 2B). The ratio of *ds-IL6R* transcript to the *fl-IL6R* isoform was small (approximately 0.03 in Asp/Asp homozygotes), as determined by normalizing the expression of the *ds-IL6R* to the *fl-IL6R* isoform (data not shown). These data indicate that the skipping of exon 9 is a rare event, which explains why the expression of the *fl-IL6R* is not affected by genotype at rs2228145. Neither of the other two SNPs in *IL6R* that showed an independent association with circulating sIL-6R were associated with *ds-IL6R* expression, after accounting for rs2228145 (Table S4). However, considering the relatively small effect sizes for the association of these two SNPs with circulating sIL-6R, our study is likely to be underpowered to detect mRNA expression effects secondary to rs2228145.

The strong effect of rs2228145 in the regulation of circulating sIL-6R has led to the hypothesis that this is the functional mechanism underlying the disease association of this variant. However, very little is known about the potential role of this genetic variant in the regulation of surface expression of IL-6R at the protein level, particularly in immune cell subsets expressing high levels of IL-6R, and whether this could affect classical IL-6R signaling. As 358Ala did not affect the expression of membrane-bound IL-6R at the mRNA level, we next assessed the effect of this variant on the expression of surface IL-6R in individual cells. We used polychromatic flow cytometry to measure the surface expression of IL-6R in PBMCs from 128 Cambridge BioResource donors (Table S5) on the four immune cell subsets of PBMCs which we found to express IL-6R (Figure S2): CD4+ naïve and memory T cells, regulatory T cells (Tregs) and monocytes.

**Figure 3:**
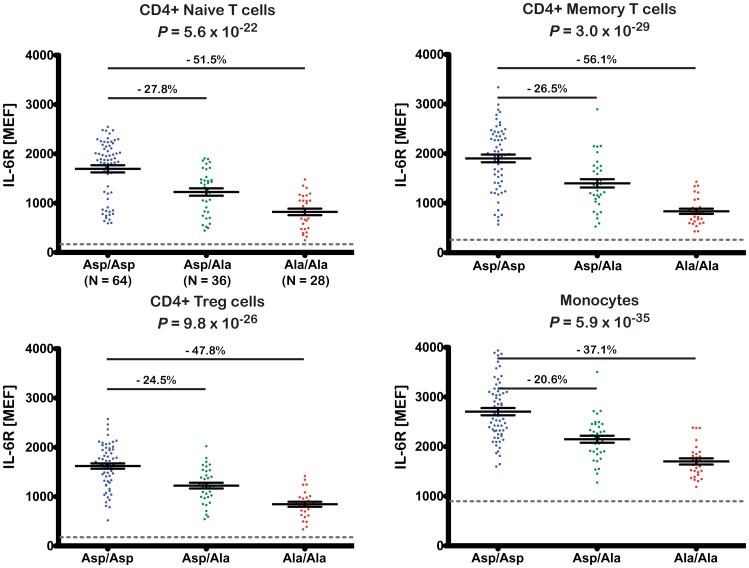
The 358Ala allele is associated with decreased levels of membrane-bound IL-6R. Surface expression of IL-6R was quantified by flow cytometry in cryopreserved PBMCs from 128 volunteers from the Cambridge BioResource. Donors were sampled according to rs2228145 genotype. IL-6R surface expression was measured in four distinct immune cell subsets: CD4+ naïve and memory T cells, CD4+ regulatory T cells (Treg) and monocytes. Scatter plots depict the individual normalized IL-6R fluorescence intensity values measured as molecules of equivalent fluorochrome (MEF; see Methods for details). Error bars represent the standard error of the mean as shown by the middle horizontal line. The horizontal grey dotted reference line represents the average background fluorescence signal of the isotype control group. Differences in the mean expression levels, relative to the common homozygote group (Asp/Asp) are indicated above the horizontal black lines. *P*-values represent test for an association of rs2228145 with surface IL-6R levels, using an additive allelic effects model (see Methods for details).

The 358Ala allele was strongly associated with reduced surface levels of IL-6R on all four immune cell subsets (*P*≤5.6×10^−22^; Figure 3). The effect sizes across the different cell types equated to a per-allele reduction of approximately 20.6–27.8% (Figure 3). To exclude the possibility that the observed results arose from a technical artifact caused by an altered epitope induced by or in linkage disequilibrium (LD) with rs2228145, we confirmed IL-6R measurements using a different anti-IL-6R antibody clone (Figures S3 and S4 and Methods). As expected, we found no evidence of genotype-specific differences on the surface expression of the gp130 co-receptor (Figure S5).

**Figure 4:**
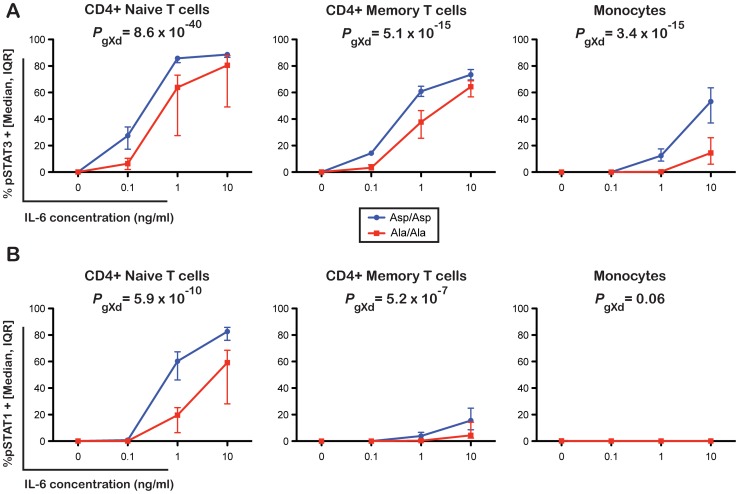
The 358Ala allele is associated with reduced IL-6 signaling potential. (A) Frequency of pSTAT3 and (B) pSTAT1 positive cells following stimulation of PBMCs with 0, 0.1, 1 or 10 ng/ml of IL-6. Intracellular levels of pSTAT3 and pSTAT1 were measured by flow cytometry in three distinct immune cell subsets: CD4+ naïve T cells, CD4+ memory T cells and monocytes in 14 Asp/Asp and 14 Ala/Ala volunteers from the Cambridge BioResource. Median and interquartile range of the distribution of the frequency of pSTAT3 and pSTAT1 positive events in the two genotype groups for each dose of IL-6 stimulation are plotted. *P*-values represent tests for differences between rs2228145 genotype groups in pSTAT activation compared to control across doses. (see Methods and Figure S6 for details).

Given the reduction of membrane-bound IL-6R expression in 358Ala carriers, we hypothesized that 358Ala would impair classical IL-6 signaling. We quantified the proportion of cells phosphorylating STAT3 and STAT1 in response to IL-6 stimulation in a subset of 14 Asp/Asp and 14 Ala/Ala homozygous donors (Table S6). While there was no significant difference between genotype groups in the absence of IL-6, carriers of 358Ala showed a significantly lower frequency of pSTAT activation upon IL-6 stimulation in the three assessed cell types: CD4+ naïve T cells (*P*_gXd_(pSTAT3) = 8.6×10^−40^, *P*_gXd_(pSTAT1) = 5.9×10^−10^), CD4+ memory T cells (*P*_gXd_(pSTAT3) = 5.1×10^−15^, *P*_gXd_(pSTAT1) = 5.2×10^−7^) and monocytes (*P*_gXd_(pSTAT3) = 3.4×10^−15^) (Figure 4; for modeled mean differences see Figure S6). The intracellular immunostaining method did not allow Treg discrimination (see Methods). Monocytes were somewhat less sensitive to IL-6 stimulation than the other two cell types, and did not show any noticeable dose response effect with pSTAT1 or genotype-dependent differences (*P* = 0.06). Qualitatively similar results were observed when assessing the MEF values of pSTAT3 and pSTAT1 induced in the three cell populations (Figure S7). In addition, we found a strong correlation between the surface levels of IL-6R and pSTAT3 activation at the dose found to have the strongest genotype-dependent differences in pSTAT3 activation in response to IL-6 stimulation (R^2^>0.7 in CD4+ naïve and memory T cells and R^2^ = 0.6 in monocytes; Figure S8). Further supporting the specificity of surface levels of IL-6R on the observed pSTAT signaling differences, we found no genotype-specific effect on the activation of pSTAT3 or pSTAT1 in response to IL-27, which shares the gp130 co-receptor or on the activation of pSTAT3 in response to IL-10, which signals through a different receptor (data not shown). These data are in agreement with a recent report showing that IL-6R-mediated pSTAT3 or pSTAT1 signaling differences in patients with relapsing-remitting multiple sclerosis were not recapitulated following stimulation with IL-10 or IL-27 [18]. Thereby, our study corroborates and improves on recent indirect evidence, showing that the expression of IL-6 target genes after stimulation with IL-6 is associated with a proxy variant in high LD with rs2228145 [10].

Interestingly, IL-6 is known to induce the differentiation of the STAT3-dependent Th17 lineage from naïve T cells [19]–[21] and inhibit FOXP3 expression [22], [23]. In our study we did not find any evidence for an effect of rs2228145 on the homeostatic frequencies of the four cell types studied (CD4+ naïve and memory T cells, Tregs and monocytes; Table S7). However, an effect on differentiation of naïve T cells under specific Th17 polarizing and Treg inducing conditions cannot be excluded. The contrasting role of Th17 cells and Tregs in the regulation of inflammation is becoming increasingly apparent [24], and provides a hypothesis for the protective role of 358Ala in the pathogenesis of human inflammatory diseases.

## Discussion

The accumulating evidence linking the Asp358Ala non-synonymous variant with risk of multiple human diseases underscores the relevance of IL-6 signaling in the etiology of inflammatory diseases. In this study, we provide evidence for the association of the minor 358Ala allele of this variant with protection from T1D. While the association was not at a GWAS level of significance (for which 22,000 cases and controls would be required to have 80% power; Table S8) the association was replicated in two independent sample sets with consistent effect estimates as reported for RA, CHD, AF, and AAA. Furthermore, we have confirmed that the 358Ala allele is associated with an increase (of approximately 35%) in the concentrations of circulating sIL-6R and demonstrated that while rs2228145 is the major genetic determinant for this trait in the *IL6R* locus, there are two additional variants with small independent effects.

Most importantly, we provided comprehensive evidence for the molecular mechanism of the 358Ala variant. The lack of an association between 358Ala and expression of the mRNA encoding the membrane-bound form of IL-6R, combined with the allele's strong association with reduced surface IL-6R expression at the protein level, suggests that 358Ala exerts an effect on proteolytic cleavage of the membrane-bound receptor independent of its effect on alternative splicing. Increased shedding of surface IL-6R in carriers of the 358Ala allele could be interpreted as a mechanism to dampen chronic classical IL-6 signaling and prevent exacerbated IL-6-driven inflammation, which is concordant with our results.

Our findings that 358Ala regulates surface expression of IL-6R were in contrast to a previous study using lymphoblastoid cell lines [12], which did not find differences in surface IL-6R expression according to rs2228145. There are, however, several potential reasons for this discrepancy. Firstly, while we determined IL-6R surface expression in specific cell subtypes expressing high levels of IL-6R, the previous study was performed on EBV-transformed B-cell lymphoblastoid lines, with very low or no expression of IL-6R, which are therefore unlikely to show any genotype dependent differences. Secondly, whilst our study used primary cells, which were frozen directly after collection, the previous study employed immortalized cell lines. Cell culturing and immortalization processes are likely to have influenced expression of the IL-6R and potentially also enzymes required for proteolytic cleavage of IL-6R. This was supported by our observation that culturing PBMCs, even for a very short period reduces surface expression of IL-6R (data not shown).

Thus, we have provided the first clear evidence that the common non-synonymous variant rs2228145 regulates the balance of surface and sIL-6R, and also affects the responsiveness of immune cells to IL-6 stimulation. This mechanism underpins the effect of 358Ala on the IL-6/IL-6R pathway and has implications for our understanding of the role of IL-6 signaling in the etiology of human disease and therapeutic targeting of this signaling pathway.

Inflammation has been implicated in the etiology of RA, AF, AAA, T1D and CHD, including reports of higher circulating levels of IL-6 preceding the onset of some of these conditions [25], [26]. 358Ala is associated with higher circulating IL-6 levels [5], [6], [12], [27], but yet protective in these diseases. This apparent paradox may be explained in two ways. Firstly, given our finding of decreased IL-6R surface expression and signaling with carriage of 358Ala, it is likely that the increase in circulating IL-6 is an indirect effect resulting from reduced IL-6 clearance through membrane-bound IL-6R in the liver [28]. This interpretation is concordant with the observation of lower circulating levels of C-reactive protein [5], [6], [29] and fibrinogen [5], [6], [30], [31], liver-derived markers of systemic inflammation, in carriers of 358Ala. Secondly, in the circulation, sIL-6R and sgp130 are in molar excess over IL-6 and any secreted IL-6 will therefore bind to sIL-6R, which will subsequently bind to sgp130, rendering IL-6 inactive [32]. The 358Ala associated increase in sIL-6R will therefore lead to an increased IL-6 buffering capacity of the circulation, thus explaining the increase in (inactive) IL-6 levels, as observed for 358Ala. Therefore, it is likely that the observed reductions in IL-6 classic signaling in carriers of 358Ala in this study may be even more pronounced under physiological conditions, where IL-6 can be inactivated by the sIL-6R/gp130 buffer system. Interestingly, it has been shown that monocytes isolated from long-term T1D patients spontaneously produce increased concentrations of IL-6, which has been linked to the differentiation of pathogenic Th17 cells [33]. Under these conditions, reduced classical IL-6 signaling could protect from chronic inflammation and differentiation of Th17 cells, consistent our finding of reduced risk of T1D in carriers of the 358Ala allele.

In contrast to the strong effect of 358Ala on classical IL-6 signaling, the effect of this variant on trans-signaling is less clear, since the nature of the samples available for this study did not enable us to investigate trans-signaling. Although we confirmed that 358Ala increases the levels of the trans-signaling mediator sIL-6R in the circulation, a material effect on systemic trans-signaling activity is unlikely, since the vast majority of the IL-6/sIL-6R complexes will be bound to the natural inhibitor sgp130 [3], [32] and, therefore, be inactive. This is consistent with the protective effect of 358Ala in RA, which mirrors the clinical effect of tocilizumab, an anti-IL-6R agent that blocks both classic and trans-signaling [34]. However, the effects of 358Ala on sIL-6R generation may be relevant in the local context of specific tissues. In the lung, for example, experimental evidence in mice suggests distinct roles for classic and trans-IL-6 signaling in the progression of airway inflammation and asthma [35]. While classic signaling appears responsible for regulatory T-cell suppression, trans-signaling seems to promote T helper 2 cell polarization in the lung. IL-6R has been shown to be expressed in the epithelium, smooth muscle and vascular endothelium of human airways, and in macrophages and granulocytes of bronchoalveolar lavage fluid (BALF) [36]. Soluble IL-6R levels in BALF are elevated in asthmatic patients compared to controls, and are elevated further upon allergen challenge [35], indicating an important role of sIL-6R in the context of the lung and associated tissues. Consistent with these findings, 358Ala is also associated with an increased severity of asthma [36]. Future studies examining the effects of this variant on the lung and associated tissues are therefore warranted.

More generally, as our findings support a causal role for classical IL-6 signaling in CHD, they intensify interest in recently-launched phase III trials of anti-inflammatory agents in the secondary prevention of CHD [37]–[40]. Furthermore, our findings, in conjunction with genetic associations with RA and CHD, and now T1D at the same locus, suggest IL-6 signaling pathways as a potential mechanistic link between these conditions, with immediate clinical relevance given the increased risk of CHD in RA [41], [42] and T1D patients [43], [44].

Our findings have established that a common variant in *IL6R*, previously known to be associated with several common complex diseases, has specific and important effects on IL-6 signaling. As the IL-6 pathway is a major therapeutic target for several human diseases [45], [46], our findings should inform the clinical development of IL-6 inhibitors and encourage exploration of pharmacogenetic or stratified medicine approaches that exploit common functional genetic variation in *IL6R* to optimize targeting and dosing of such agents in people with different genetic profiles.

## Methods

### Subjects

All T1D patients were under 17 years of age at diagnosis and recruited from across Great Britain and have been described previously [47]. Controls were obtained from the British 1958 Birth Cohort and the Wellcome Trust Case-Control Consortium UK Blood service Common Control (UKBS) sample collection [48], [49] both of which were recruited from across Great Britain and geographically matched to cases in tests for association with T1D.

All 3,771 T1D families were of white European decent. 403 multiplex (affected sibling pair) families were from the Diabetes UK Warren I collection; 43 simplex families from Yorkshire, Great Britain; 211 multiplex/simplex families from Northern Ireland; 275 multiplex families were from the Human Biological Data Interchange; 956 multiplex/simplex families from Finland; 215 simplex families from Romania; the remaining 1,668 multiplex families were made available through the Type 1 Diabetes Genetics Consortium.

In addition to 1,761 T1D cases and 1,030 British Birth cohort controls, 2,221 plasma samples were randomly selected from blood donors who joined the Cambridge BioResource during their local blood donation sessions at NHS Blood and Transplant, for measurement of circulating sIL-6R levels (1,034 recruited in 2007, BR4000 and 1,187 recruited in 2009, BR8000). All subjects were of white European ancestry.

### Ethics statement

All samples and information were collected with written and signed informed consent. The study was approved by the local Peterborough and Fenland research ethics committee for the project entitled: ‘An investigation into genes and mechanisms based on genotype-phenotype correlations in type 1 diabetes and related diseases using peripheral blood mononuclear cells from volunteers that are part of the Cambridge BioResource’ (05/Q0106/20).

### Genotyping

Samples for sIL-6R analysis were genotyped using the Illumina ImmunoChip platform [13]. Individuals for the T1D association analysis (8,371 T1D patients and 10,092 healthy controls), T1D family collections and all Cambridge BioResource volunteers selected for cell-based experiments were genotyped at rs2228145 using TaqMan (Applied Biosystems).

### Soluble IL-6R Europium-Streptavidin ELISA

Circulating sIL-6R concentrations were measured using a highly sensitive non-isotopic time-resolved fluorescence ELISA assay based on the dissociation-enhanced lanthanide fluorescent immunoassay technology (DELFIA; PerkinElmer). Test plasma samples diluted 1∶20 in PBS+10% FBS were measured in duplicate on 384-well MaxiSorp microtiter plates (Nunc), coated with 1 µg/ml monoclonal anti-human IL-6R antibody (clone 17506; RD Systems). Detection was performed using a biotinylated mouse anti-CD126 monoclonal antibody (clone M182, BD Biosciences) diluted to a final concentration of 100 ng/ml in PBS+10% FBS and a Europium-Streptavidin detection solution (PerkinElmer), diluted in PBS+0.05% tween, 1% BSA, 7 µg/ml DTPA to a final concentration of 0.05 µg/ml. Quantification of test samples was obtained by fitting the readings to a human recombinant IL-6Rα (RD systems) serial dilution standard curve plated in quadruplicate on each plate.

### Relative *IL6R* mRNA expression by RT–qPCR

mRNA expression was measured in 88 healthy CBR donors. cDNA was generated from total RNA isolated from peripheral blood mononuclear cells (PBMCs) as described previously [50]. Relative expression of the *fl-IL6R* and *ds-IL6R* isoforms was determined by real-time quantitative PCR (TaqMan, Applied Biosystems). We designed two specific forward primers to hybridize to unique exon boundaries of *IL6R* (*fl-IL6R*: exons 9/10; *ds-IL6R*: exons 8/10) and a common reverse primer and probe to amplify the two *IL6R* isoforms. Primers and probe sequences are summarized in Table S9. Full-length *IL6R* and *ds-IL6R* expression was normalized to the β2 microglobulin housekeeping gene. In addition, *ds-IL6R* was normalized to *fl-IL6R* to allow approximation of the *ds-IL6R*/*fl-IL6R* ratio. All probes were labeled with FAM and a non-fluorescent quencher (BHQ1; Sigma). PCR efficiency and the amplification factor for each reaction were calculated using a 1∶2 serial dilution curve of 8 random cDNA samples. The PCR efficiency was 100.2%, 94.2% and 97.3% and the resulting amplification factor was 2.0024, 1.9423 and 1.9735 for the *B2M*, *fl-IL6R* and *ds-IL6R* reactions, respectively [51]. Relative gene expression levels were calculated using the formula af∧ΔCT, with af representing the amplification factor of the respective PCR reaction and ΔCT the difference in cycle threshold between the target and control transcripts.

### Surface immunostainings

128 samples for flow cytometry experiments were selected according to rs2228145 genotype from a CBR subset with available cryopreserved PBMCs (Table S5). To reduce the effects of day-to-day variation, where possible, rare homozygotes (Ala/Ala) were matched to one or more common homozygotes and up to three heterozygotes, from the same 10-year age band and sex. Batches of up to ten samples were assembled from the matched groups, maximizing diversity of the age, sex and T1D distribution within each batch. Since there were no differences in IL-6R surface expression between T1D patients and controls, we included T1D cases in the primary analyses to increase power. Primary analyses were adjusted for T1D status, because limited availability of Asp/Asp and especially Ala/Ala T1D cases resulted in oversampling of T1D cases in the heterozygote group (Table S5). All assays were performed blinded to sample genotype and disease status.

PBMCs were isolated and cryopreserved (10×10^6^ cells/aliquot) as described previously [52]. Cryopreserved PBMCs were thawed in a 37°C water bath and resuspended in X-Vivo (Lonza)+10% heat-inactivated, filtered human AB serum (Sigma) in a drop-wise fashion and then washed in X-Vivo+1% AB serum. 5×10^5^ cells were stained for 1 h at 4°C (using the surface immunostaining Panel 1 detailed in Table S10), washed with BD CellWash (BD Biosciences) and then fixed with freshly prepared BD CellFix (BD Biosciences). To exclude the possibility that an altered IL-6R epitope induced by the Asp358Ala polymorphism could affect the binding affinity of the anti-IL6R antibody used in this study (clone UV4), we designed a second surface immunostaining panel (Panel 2; Table S10). There was a near-perfect correlation between the two anti-IL-6R clones used in this study in the three immune cell subsets that were directly comparable (Spearman rho >0.93; Figure S3). We found that measurements of IL-6R expression were reproducible between two independent measurements (Spearman rho >0.71; Figure S9).

We also performed surface staining of random PBMC donors using global lineage discrimination markers, including α-CD19, α-CD8 and α-CD56 to assess surface IL-6R and gp130 expression levels on the main immune cell subsets in addition to CD4 T cells and monocytes, which we have focused on this study.

### Intracellular pSTAT3 and pSTAT1 immunostainings

IL-6 signaling experiments employed a subset of the matched groups for the surface immunostainings, consisting of one Asp/Asp homozygote and one Ala/Ala homozygote pair from each matched group, for those where additional cryopreserved PBMC aliquots were available. All assays were performed blinded to sample genotype and disease status.

To assess IL-6 responsiveness, cryopreserved PBMCs were thawed as described above and aliquots of 5×10^5^ cells resuspended in 100 µl X-Vivo+1% AB serum, plated in a U-bottom 96-well cell culture plate (Cellstar) and rested for 10 min at 37°C before stimulation. Following stimulation with 0, 0.1, 1 or 10 ng/ml of IL-6 for 10 min (37°C, 5% CO_2_), cells were immediately fixed with BD phosphoflow lyse/fix buffer (BD Biosciences) to maintain their phosphorylation state and incubated for 10 min (37°C, 5% CO_2_). After washing with PBS+0.2% BSA, cells were permeabilized with 100%, ice cold, methanol and incubated at 4°C for 30 min. Cells were then washed with PBS+0.2%, and blocked for 15 min with PBS+1% BSA. Staining was performed as described above using the intracellular immunostaining Panel 1 (Table S10) and cells resuspended in PBS+0.2% BSA. The anti-CD127 and anti-CD25 antibodies required for Treg discrimination were not compatible with the methanol fixation and, therefore, not included in the intracellular immunostaining panel.

### Flow cytometry

Immunostained samples were analyzed using a BD Fortessa (BD Biosciences) flow cytometer with FACSDiva software (BD Biosciences). Flow cytometry data were exported in the format 3.0 and analyzed using FlowJo (Tree Star, Inc.). Gating strategy was performed as depicted in Figure S2. Doublet exclusion was performed for both CD4+ T cell and monocyte populations. Cyto-Cal calibration beads (Thermo Scientific) were used to assess instrument stability and to convert individual mean fluorescence intensity (MFI) values into normalized molecules of equivalent fluorochrome (MEF) values as described previously [53]. Distribution of the unstained (FMO; fluorescence minus one), isotype control and stained test samples using the anti-IL-6R UV4 clone (surface immunostaining Panel 1) or anti-gp130 antibodies (surface immunostaining Panel 2) are depicted in Figure S10.

### Statistical analyses

Statistical analyses were performed using Stata (www.stata.com), regional association plots were produced by LocusZoom [54].

#### T1D association analysis

All SNPs were in Hardy-Weinberg equilibrium in controls (*P*>0.01). Association with T1D was analyzed in a logistic regression model with disease status as outcome variable, and counts of the SNP minor allele as independent variable (i.e. assuming a multiplicative allelic effects model on the odds ratio scale). Cases and controls were stratified according to 12 broad geographical regions to allow for variation in allele frequency and disease incidence across the United Kingdom. Families were analyzed by generating cases and matched pseudo-controls and analyzing them with conditional logistic regression [55]. A multiplicative allelic effects model on the odds ratio scale was assumed.

#### Association analysis of circulating sIL-6R

To ensure normality, circulating sIL-6R was log_10_ transformed for all analyses. Each collection was tested for batch effects of the sIL-6R assay and potential covariates (Table S1). Linear regression was used with log_10_(sIL-6R) as outcome and covariates as independent variables. No between-collection variability in log_10_(sIL-6R) remained once within-collection batch effects were accounted for. Association of log_10_(sIL6R) with SNPs in the *IL6R* region was tested in a regression model with log_10_(sIL6R) as dependent variable and SNP, assuming an additive allelic effects model, as independent variable. All necessary batch effects were included. Significance was assessed with a likelihood ratio test and *P*<5×10^−8^, a typical genome-wide significance threshold, was considered significant. Validity of the additive effects model was verified by comparison with a model that assumed no specific mode of inheritance using a likelihood ratio test and this model was used in preference to the additive effects model, where suggestive evidence of deviation from additivity was obtained. Additional independent SNP effects were tested in a forward stepwise regression with use of a likelihood ratio test and *P*<1×10^−4^ was considered significant.

#### Analysis of mRNA and surface IL-6R expression with rs2228145

Using Levene's robust test for the equality of variances between groups applied to the residuals, the mRNA expression data was found to have unequal variances by genotype group *i.e.* they were heteroscedastic (*P*<0.05). Therefore, inferences based on ordinary linear regression could be biased. Consequently, analysis of the mRNA expression data was performed using a multilevel mixed-effects linear regression model with expression (relative to *B2M* or *fl-IL6R*) as the dependent variable and rs2228145 genotype as the independent variable, but estimating the residual errors for each individual genotype group separately. This allows for different variability of mRNA expression for each genotype group. Storage plate was included as a covariate.

Similarly, surface IL-6R expression, could not be analyzed using ordinary linear regression as individuals were sampled according to genotype group (non-random sampling) and found to have unequal variances across genotype groups using Levene's test, *P*<0.05. Therefore a multilevel mixed-effects linear regression model was adopted, with surface IL-6R expression (MEF) as dependent variable and rs2228145 genotype as the independent variable, again estimating the residual errors for each individual genotype group separately. Age (coded in 10-year bands), sex, T1D status, and measurement batch were included as covariates.

*P*-values for genotype effects (for both mRNA and surface expression) correspond to those of an additive allelic effects model of inheritance. The model residuals were normally distributed, and the additivity assumption held (tested by comparing the allelic effects test with a model that assumed no specific mode of inheritance).

#### Analysis of IL-6 stimulation with Asp358Ala

The data from the cytokine (IL-6) stimulation experiments is measured on the cellular level (*i.e.* number of pSTAT positive cells), but the experimental sampling units were the individuals and consequently, each individual has repeated measures (at different concentrations of IL-6). Observations are likely to be correlated within individuals (hierarchical), and these within-subject correlations needed to be accounted for in the analysis. Therefore, the number of pSTAT positive cells in response to cytokine stimulation was modeled using a multilevel mixed-effects logistic regression model. We were interested in the proportion of cells responding to cytokine stimulation measured by pSTAT phosphorylation. Therefore, proportion of pSTAT positive cells to the proportion of pSTAT negative cells in the parent population was used as the dependent variable in the regression model. Genotype and cytokine dose (modeled as fixed effect) were included as dependent variables in the logistic regression model. Cytokine dose was also included as a random effect to account for the within subject correlation. This allowed us to test genotype dependent differences in the control group and to model the IL-6 dose-response effect of cytokine stimulation, respectively. To test for genotype dependent differences in response to cytokine stimulation interaction terms for genotype and cytokine dose were added to the regression model as independent (fixed effects) variables and their effect on the primary IL-6 stimulation experiments (Figure 4) assessed using a Wald test. The test reflects differences between genotype groups in pSTAT activation compared to control across doses. The variances and covariances were distinctly estimated across cytokine doses.

*P*<0.004 was considered significant (α = 0.05 Bonferroni corrected for 12 tests, two mRNA transcripts, four cell types for surface staining, three (cell types)×two (STAT proteins)).

### Assay characteristics and variation

For the sIL-6R assay, the average CV between duplicate samples was relatively consistent for each batch, and averaged 8.58% in the entire study (samples with CV>15% were excluded from the analyses). Technical repeatability was examined by measuring the same 16 samples within each of the 6 batches. The average CV for the 16 repeats performed in each batch was 8.1% (range 6.86% to 20.81%), which suggests these results were as consistent as duplicate samples within the same plate (hence no obvious batch effects). An average R^2^ of 0.9995 (with a range of 0.9975 to 1) was determined for the standard curves across all batches. The minimum and maximum detectable concentrations within the linear range were given as 4.69 ng/ml and 300 ng/ml respectively. All measured plasma samples were within these values, with a minimum of 8.3 ng/ml and a maximum of 133.4 ng/ml.

For qPCR measurements, test samples were measured in duplicate, with an average intra-assay CV% of 0.97%, 0.67% and 0.78% between replicates for the *B2M*, *fl-IL6R* and *ds-IL6R* reactions, respectively (data not shown). The flow cytometer was found to be very stable during the entire experimental procedure for both the PE and APC quantification channels (average CV% for the five fluorescent bead populations = 1.77% and 4.63%, respectively).

### Anti-IL-6R competition assays

For the anti-IL-6R competition assays, aliquots of 5×10^5^ PBMCs from a donor with high levels of surface IL-6R expression were resuspended in 100 µl X-Vivo+1% AB serum and plated in a U-bottom 96-well cell culture plate (Cellstar). Cells were incubated with 0, 0.01, 0.1 or 1 µg of unconjugated anti-IL-6R UV4 antibody, unspecific mouse IgG1κ control, or with 1 µg of the unconjugated 17506 anti-IL-6R clone used in the ELISA assays for 1 h at 4°C. Cells were then washed with PBS+0.2% BSA (Sigma) and stained using either the surface immunostaining Panel 1 or Panel 2 as described above. The unconjugated UV4 monoclonal antibody (mAb) was unable to block binding of labeled BL-126. Conversely, the anti-IL-6R clone (17506), used to measure sIL-6R, only inhibited BL-126 staining (Figure S3), showing that both clones bind to the same or adjacent epitopes. Since Ala358 was associated with increased concentrations of sIL-6R measured by ELISA using the 17506 mAb, these data demonstrate that, not only the UV4 and BL-126 clones are recognizing different epitopes, but also that the genotype-specific differences in IL-6R expression could be not be due to structural differences affecting antibody affinity caused by Asp358Ala.

## Supporting Information

Figure S1 Association of rs2228145 with human diseases. Odds ratios (OR) and 95% confidence intervals (CI) are given for the reported associations of rs2228145 with human diseases. Box sizes are proportional to the number of cases for each effect estimate. ^*^Analysis based on proxy variants (r^2^>0.96 with rs2228145); ^†^Respectively: Relative Risk (95% CI), Number of affected offspring, Total number of families.
(TIF)

Figure S2 Gating strategy for the immune cell subsets. (A) Depicted is the gating strategy used to measure IL-6R expression using the IL-6R UV4 clone immunophenotyping panel (see Methods for details). Initially, lymphocytes and monocytes were broadly discriminated based on the forward (FSC-A) and side-scatter (SSC-A) profile. CD4+ T cells were then gated based on their expression of IL-7R (CD127) and IL-2RA (CD25) to identify the CD4+ regulatory T cell (Treg) subset (CD127^lo^ CD25^hi^). CD127^int-hi^ CD25^lo^ cells (Non-Tregs) were then further subdivided according to their surface expression of CD45RO to define the CD4+ naïve (CD45RO^lo^) and CD4+ memory (CD45RO^hi^) T cell subsets. Monocytes were gated based on their surface expression of CD14. (B) Overlaid histograms depicting the surface IL-6R staining from two illustrative donors (one 358Asp homozygote – blue histograms – and one 358Ala homozygote – red histograms) in CD4+ naïve T cells and monocytes. The two selected donors had IL-6R surface expression closest to the mean of the respective genotype group. The black and grey histograms represent the IL-6R unstained (fluorescence minus one - FMO) and PE isotype control staining profiles, respectively.
(TIF)

Figure S3 Anti-IL-6R UV4 and 17506 monoclonal antibodies recognize different IL-6R epitopes. (A) Surface IL-6R staining using the anti-IL-6R UV4 clone (standard, used for main experiments) was inhibited in a dose-dependent manner in CD4+ naïve, memory and Treg cells by pre-incubation of cells with the unconjugated anti-IL-6R UV4 blocking antibody, but not with the unconjugated anti-IL6R 17506 blocking antibody or an unspecific mouse IgG1κ control. (B) Surface IL-6R staining using the anti-IL-6R BL-126 clone was unaffected by the UV4 clone, but was blocked by the 17506 clone. MFI, mean fluorescence intensity; mAB, monoclonal antibody.
(TIF)

Figure S4 Surface IL-6R measurements are highly correlated using two different anti-IL-6R clones. Surface IL-6R levels were measured in all 128 samples with two different anti-IL-6R antibody clones. Panels show IL-6R surface expression measurement in three different cell types, measured with the UV4 clone (x-axis, as used for main analyses) or the BL-156 clone (y-axis). Correlation coefficients (Spearman's rho) of 0.93–0.96 indicate very good correlation between the relative ordering of each sample within the two different measurements.
(TIF)

Figure S5 Expression of the gp130 co-receptor is not affected by rs2228145 genotype. Surface expression of the gp130 co-receptor was quantified by flow cytometry in cryopreserved PBMCs from 128 volunteers from the Cambridge BioResource. Sampling of donors was stratified by genotype at rs2228145 and IL-6R expression was measured in four distinct immune cell subsets: CD4+ naïve and memory T cells, CD4+ regulatory T cells (Treg) and monocytes. Scatter plots depict the individual normalized gp130 fluorescence intensity values measured as molecules of equivalent fluorochrome (MEF). Error bars represent the standard error of the mean as shown by the middle horizontal line. The horizontal grey dotted reference line represents the average background fluorescence signal of the isotype control group. *P*-values represent tests for an association of rs2228145 with surface gp130 levels, using an additive allelic effects model (see Methods for details).
(TIF)

Figure S6 Differences between genotypes in %pSTAT-positive cells (as derived from regression model) Mean difference and 95% CIs (error bars) in pSTAT3 (A) and pSTAT1-positive cells (B) between genotype groups (Ala/Ala – Asp/Asp) are plotted against IL-6 concentrations. These differences and *P*-values correspond to the data displayed in Figure 4. The Ala/Ala genotype group is used as the reference. Therefore, negative differences indicate a lower proportion of pSTAT positive cells in the Ala/Ala homozygotes, compared to Asp/Asp (common) homozygotes at the given IL-6 concentration. The *P*_gxd_ are for testing whether differences between genotypes vary according to IL-6 dose, thus informing whether the response to IL-6 stimulation is modified by genotype (*i.e.* whether there is a statistical interaction between genotype and IL-6 concentration; see Methods).
(TIF)

Figure S7 358Ala is associated with decreased sensitivity to IL-6 signaling. Intracellular levels of pSTAT3 (A) and pSTAT1 (B) following stimulation of PBMCs with 0, 0.1, 1 or 10 ng/ml of IL-6. Activation of pSTAT3 and pSTAT1 was measured by flow cytometry in three distinct immune cell subsets, CD4+ naïve T cells, CD4+ memory T cells and monocytes in 14 358Asp and 14 358Ala homozygous volunteers from the Cambridge BioResource. Values represent the median and interquartile range (IQR) of the distribution of the fluorescence intensity values of pSTAT3/pSTAT1, measured as molecules of equivalent fluorochrome (MEF), in the two genotype groups for each dose of IL-6 stimulation.
(TIF)

Figure S8 pSTAT3 activation is dependent on the surface levels of IL-6R. Correlation between surface levels of IL-6R and phosphorylated levels of intracellular STAT3 following stimulation with 0 ng/ml (depicted in black) or 1 ng/ml (depicted in blue) of IL-6, which was the dose where we found the strongest genotype-dependent effect of Asp358Ala on pSTAT3 activation. Intracellular levels of pSTAT3 were measured in CD4+ naïve and memory T cells and in monocytes from 14 Asp/Asp and 14 Ala/Ala homozygotes. R^2^, coefficient of determination; MEF, molecules of equivalent fluorochrome.
(TIF)

Figure S9 Repeatability of IL-6R measurement. In 28 donors, a second PBMC aliquot was measured again, approximately 1.5 months after the first measurement. (A) IL-6R expression from the first measurement (x-axis) versus the second measurement (y-axis). Correlation coefficients (Spearman's rho) of 0.71–0.89 indicate good correlation between the relative ordering of samples for two independent measurements. (B) Average of the two measurements, against the difference in measurements (2^nd^ measurement – 1^st^ measurement), together with the mean difference (solid line) and the limits of agreement (dashed lines) (Bland-Altman plot). On average the second measurement is lower than the first measurement, but there is no evidence for systematic bias.
(TIF)

Figure S10 IL-6R and gp130 surface immunostainings distribution. Box and whisker plots depicting the distribution of the IL-6R (A) and gp130 (B) surface staining profiles on the unstained (FMO), isotype control and test sample groups. Error bars represent the 10–90 percentiles of the distribution. MEF, molecules of equivalent fluorochrome; FMO, fluorescence minus one.
(TIF)

Table S1 Characteristics of the 4,249 individuals in whom circulating sIL-6R were measured. 3,605 samples were genotyped at 45 SNPs in the *IL6R* locus using the Illumina ImmunoChip. Mean (min-max) age-at-diagnosis of T1D cases (GRID) was 7.6 (0.6–16) years. Age = the age of the individual when the sample was taken. Note, there were plate effects (BR8000, *P* = 3.4×10^−4^) or box effects (GRID, *P* = 4.9×10^−77^; UKBS, *P* = 2.5×10^−5^; BR4000, *P* = 2.6×10^−13^), age effects (UKBS, *P* = 9.2×10^−5^; GRID, *P* = 0.0056) and for GRID, a dependence on the coefficient of variation (*P* = 7.6×10^−4^) on circulating sIL6R. GB, Great Britain; GRID, Genetic Resource Investigating Diabetes, UK BS, United Kingdom Blood Service.
(DOCX)

Table S2 Association of sIL-6R with the *IL6R* region, in 3,605 samples. b = regression coefficient for a model containing all three SNPs. se = standard error of b. R^2^ (diff) = the difference in the square of the correlation coefficient for a model with and without the listed SNP. *P* is for the addition of the listed SNP to a model containing the other two SNPs.
(DOCX)

Table S3 Characteristics of samples for qPCR expression analyses. *P*: Fisher's exact test.
(DOCX)

Table S4 Association of *IL6R* SNPs with *ds-IL6R* expression, after accounting for rs2228145 in a subset (n = 75) of individuals with information on all three *IL6R* SNPs. Betas and *P*-values correspond to an additive model of inheritance (1 df test, 2 = effect allele) with rs2228145 plus one of the other SNPs (rs4329505 or rs1386821) in the model (columns: SNP, Beta_SNP_ and *P*_SNP_).
(DOCX)

Table S5 Characteristics of samples for IL-6R surface expression. Samples have been selected and matched based on rs2228145 genotype (see methods for details). *P*-values reflect tests for differences across columns (Fisher's exact test). *P*: Fisher's exact test.
(DOCX)

Table S6 Characteristics of samples for IL-6 stimulation. Samples have been selected and matched based on rs2228145 genotype (see methods for details). *P*: Fisher's exact test.
(DOCX)

Table S7 Cell population frequencies of parent population according to rs2228145 genotype. Samples have been selected and matched based on rs2228145 genotype (see methods for details). ^*^ *P*-value derived from linear regression model, adjusting for sex, age (10-year bands), T1D status, and batch the sample was measured in.
(DOCX)

Table S8 Power calculations for the association of rs2228145 with T1D. Calculations were performed assuming a multiplicative model and an effect size (odds ratio) of 0.92, as estimated in our population of unrelated T1D patients (n = 8,371) and controls (n = 10,092).
(DOCX)

Table S9 Primers and probes sequences.
(DOCX)

Table S10 Anti-human antibodies used for flow cytometry.
(DOCX)
