# Supplementary figures and images for: Functional IL6R 358Ala Allele Impairs Classical IL-6 Receptor Signaling and Influences Risk of Diverse Inflammatory Diseases
Source: PLoS Genet. 2013 Apr 4;9(4):e1003444. doi: 10.1371/journal.pgen.1003444 (PMC3617094; doi:10.1371/journal.pgen.1003444)

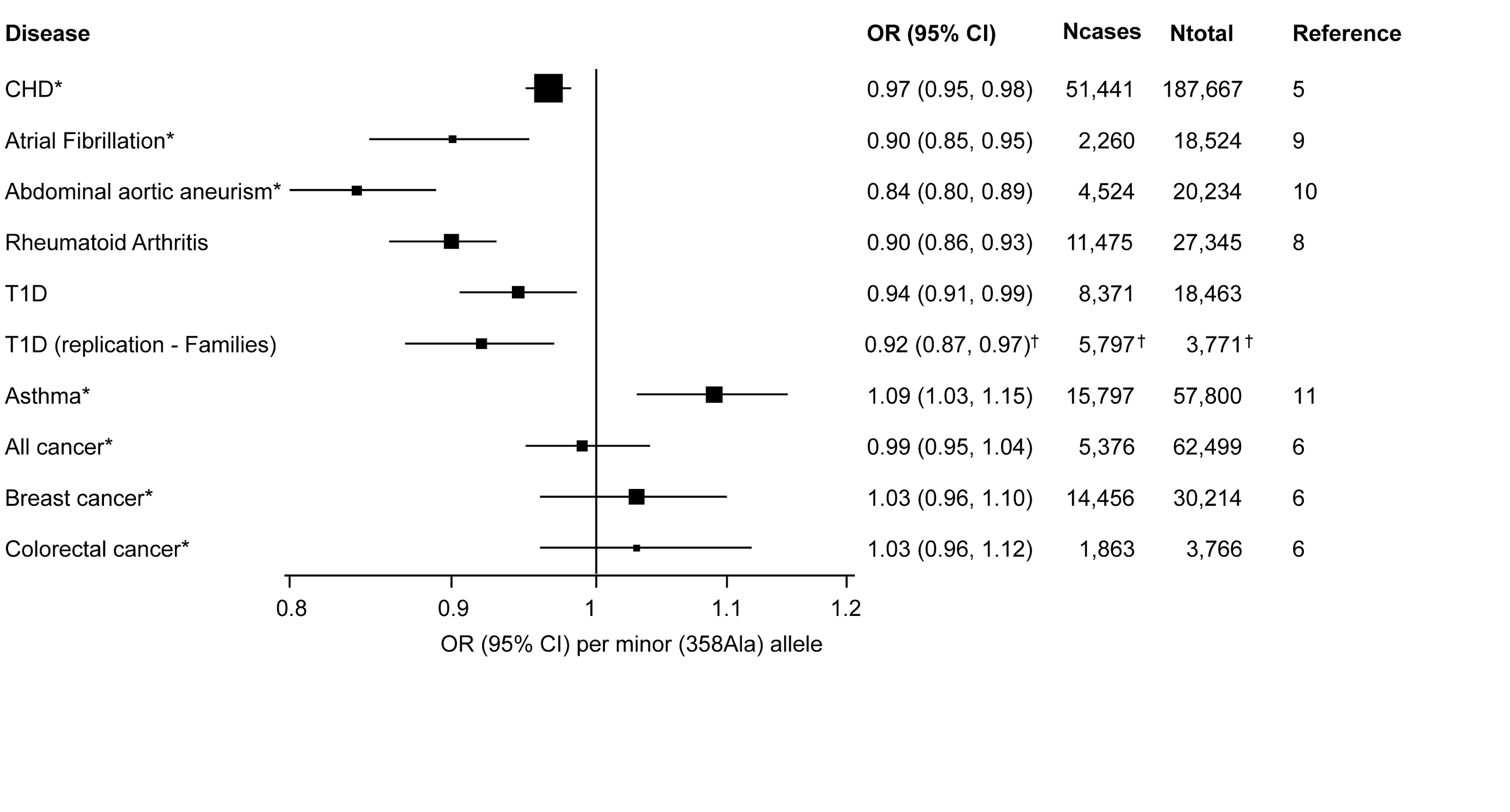

Supplement: Figure S1 — Association of rs2228145 with human diseases. Odds ratios (OR) and 95% confidence intervals (CI) are given for the reported associations of rs2228145 with human diseases. Box sizes are proportional to the number of cases for each effect estimate. *Analysis based on proxy variants (r2>0.96 with rs2228145); †Respectively: Relative Risk (95% CI), Number of affected offspring, Total number of families. (TIF) [file pgen.1003444.s001.tif]

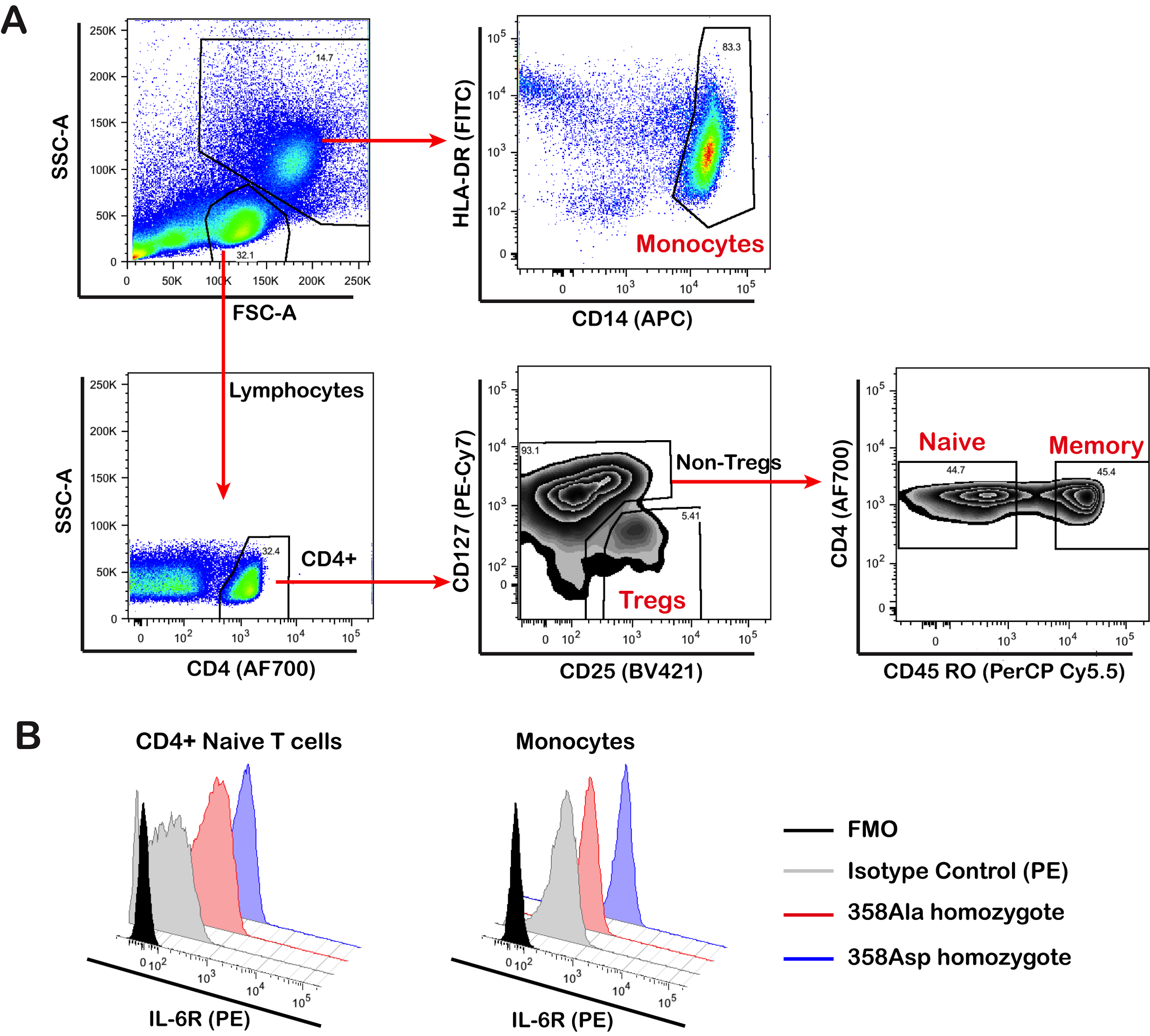

Supplement: Figure S2 — Gating strategy for the immune cell subsets. (A) Depicted is the gating strategy used to measure IL-6R expression using the IL-6R UV4 clone immunophenotyping panel (see Methods for details). Initially, lymphocytes and monocytes were broadly discriminated based on the forward (FSC-A) and side-scatter (SSC-A) profile. CD4+ T cells were then gated based on their expression of IL-7R (CD127) and IL-2RA (CD25) to identify the CD4+ regulatory T cell (Treg) subset (CD127lo CD25hi). CD127int-hi CD25lo cells (Non-Tregs) were then further subdivided according to their surface expression of CD45RO to define the CD4+ naïve (CD45ROlo) and CD4+ memory (CD45ROhi) T cell subsets. Monocytes were gated based on their surface expression of CD14. (B) Overlaid histograms depicting the surface IL-6R staining from two illustrative donors (one 358Asp homozygote – blue histograms – and one 358Ala homozygote – red histograms) in CD4+ naïve T cells and monocytes. The two selected donors had IL-6R surface expression closest to the mean of the respective genotype group. The black and grey histograms represent the IL-6R unstained (fluorescence minus one - FMO) and PE isotype control staining profiles, respectively. (TIF) [file pgen.1003444.s002.tif]

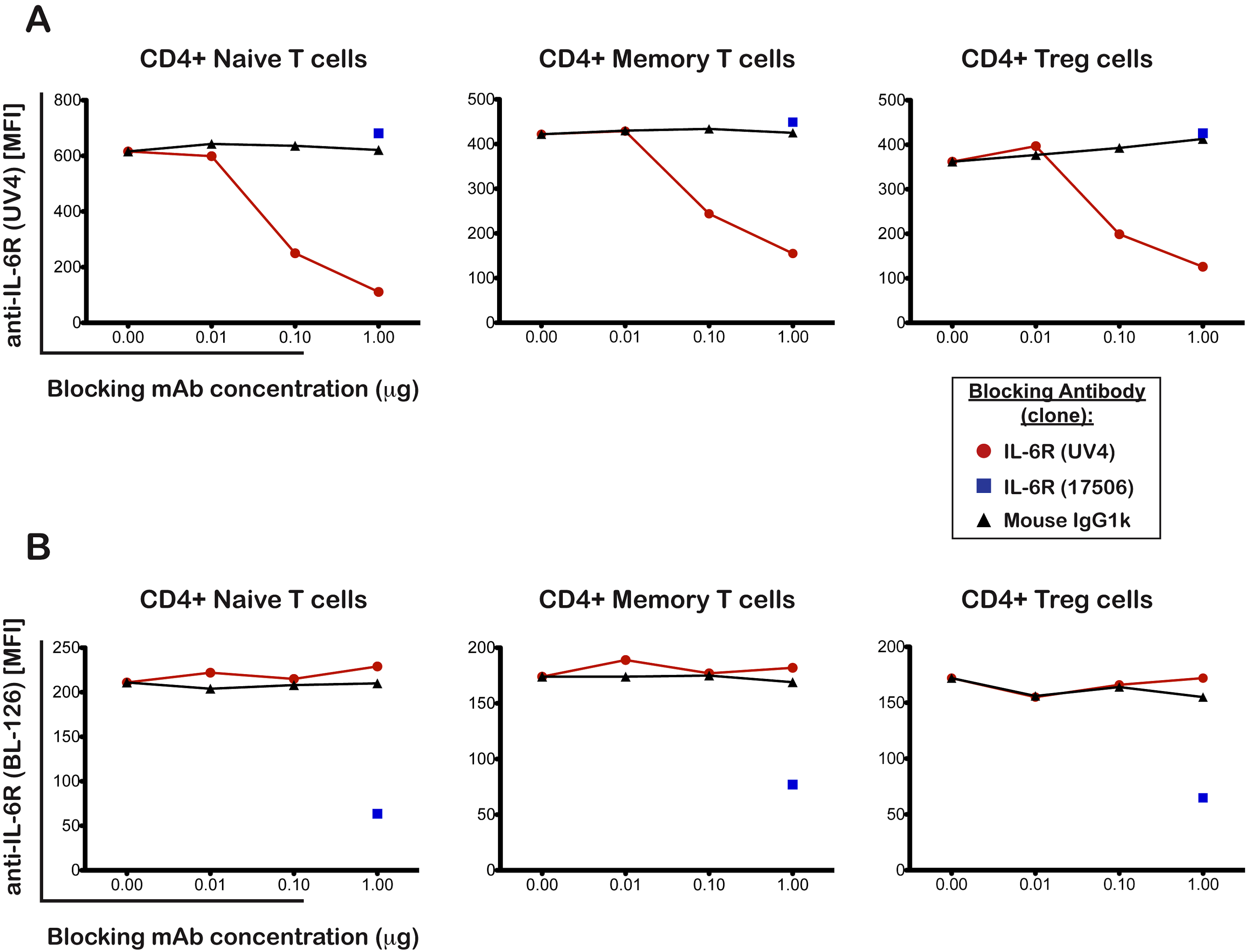

Supplement: Figure S3 — Anti-IL-6R UV4 and 17506 monoclonal antibodies recognize different IL-6R epitopes. (A) Surface IL-6R staining using the anti-IL-6R UV4 clone (standard, used for main experiments) was inhibited in a dose-dependent manner in CD4+ naïve, memory and Treg cells by pre-incubation of cells with the unconjugated anti-IL-6R UV4 blocking antibody, but not with the unconjugated anti-IL6R 17506 blocking antibody or an unspecific mouse IgG1κ control. (B) Surface IL-6R staining using the anti-IL-6R BL-126 clone was unaffected by the UV4 clone, but was blocked by the 17506 clone. MFI, mean fluorescence intensity; mAB, monoclonal antibody. (TIF) [file pgen.1003444.s003.tif]

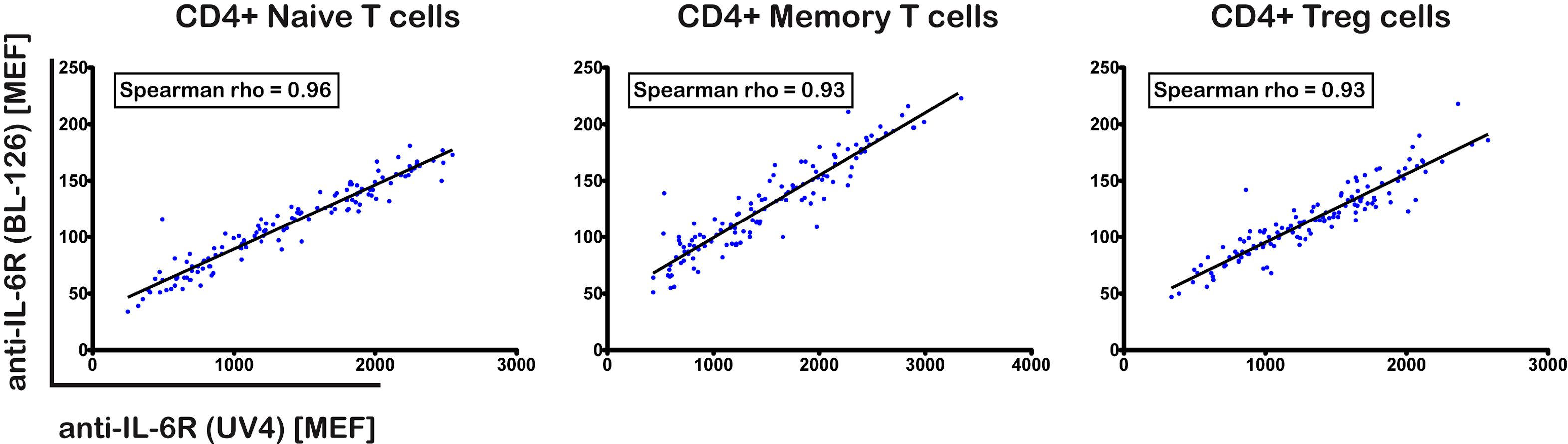

Supplement: Figure S4 — Surface IL-6R measurements are highly correlated using two different anti-IL-6R clones. Surface IL-6R levels were measured in all 128 samples with two different anti-IL-6R antibody clones. Panels show IL-6R surface expression measurement in three different cell types, measured with the UV4 clone (x-axis, as used for main analyses) or the BL-156 clone (y-axis). Correlation coefficients (Spearman's rho) of 0.93–0.96 indicate very good correlation between the relative ordering of each sample within the two different measurements. (TIF) [file pgen.1003444.s004.tif]

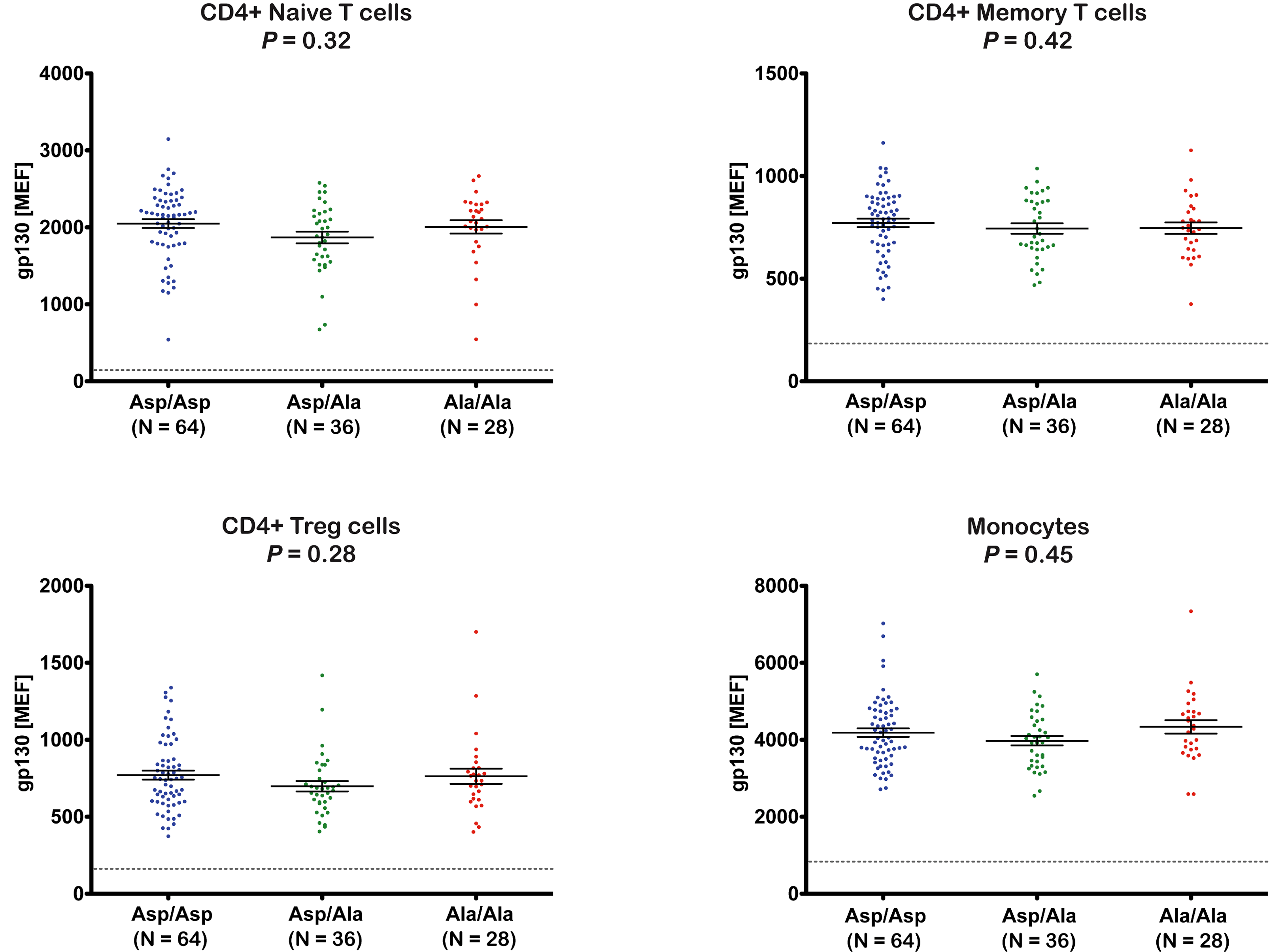

Supplement: Figure S5 — Expression of the gp130 co-receptor is not affected by rs2228145 genotype. Surface expression of the gp130 co-receptor was quantified by flow cytometry in cryopreserved PBMCs from 128 volunteers from the Cambridge BioResource. Sampling of donors was stratified by genotype at rs2228145 and IL-6R expression was measured in four distinct immune cell subsets: CD4+ naïve and memory T cells, CD4+ regulatory T cells (Treg) and monocytes. Scatter plots depict the individual normalized gp130 fluorescence intensity values measured as molecules of equivalent fluorochrome (MEF). Error bars represent the standard error of the mean as shown by the middle horizontal line. The horizontal grey dotted reference line represents the average background fluorescence signal of the isotype control group. P-values represent tests for an association of rs2228145 with surface gp130 levels, using an additive allelic effects model (see Methods for details). (TIF) [file pgen.1003444.s005.tif]

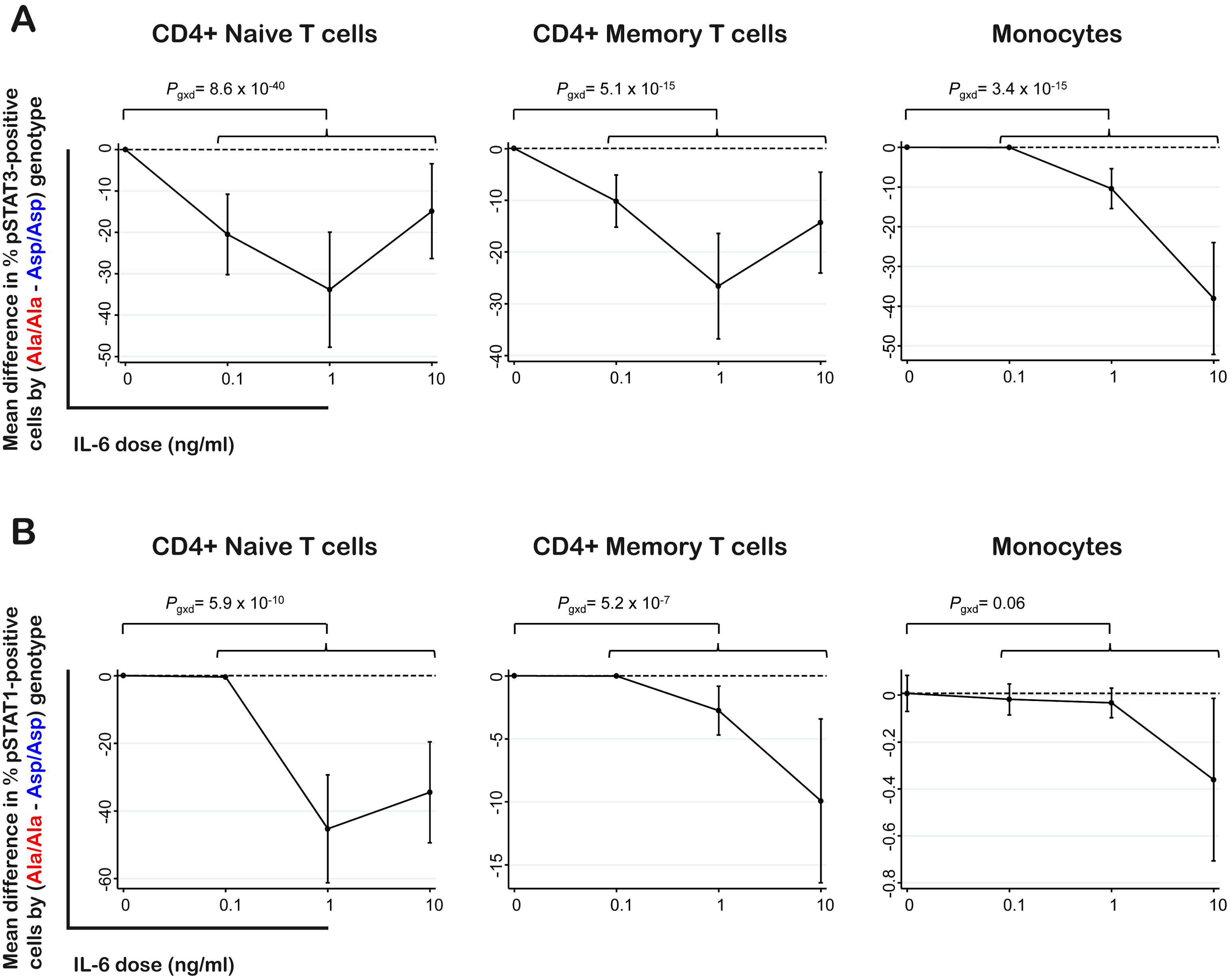

Supplement: Figure S6 — Differences between genotypes in %pSTAT-positive cells (as derived from regression model) Mean difference and 95% CIs (error bars) in pSTAT3 (A) and pSTAT1-positive cells (B) between genotype groups (Ala/Ala – Asp/Asp) are plotted against IL-6 concentrations. These differences and P-values correspond to the data displayed in Figure 4. The Ala/Ala genotype group is used as the reference. Therefore, negative differences indicate a lower proportion of pSTAT positive cells in the Ala/Ala homozygotes, compared to Asp/Asp (common) homozygotes at the given IL-6 concentration. The P gxd are for testing whether differences between genotypes vary according to IL-6 dose, thus informing whether the response to IL-6 stimulation is modified by genotype (i.e. whether there is a statistical interaction between genotype and IL-6 concentration; see Methods). (TIF) [file pgen.1003444.s006.tif]

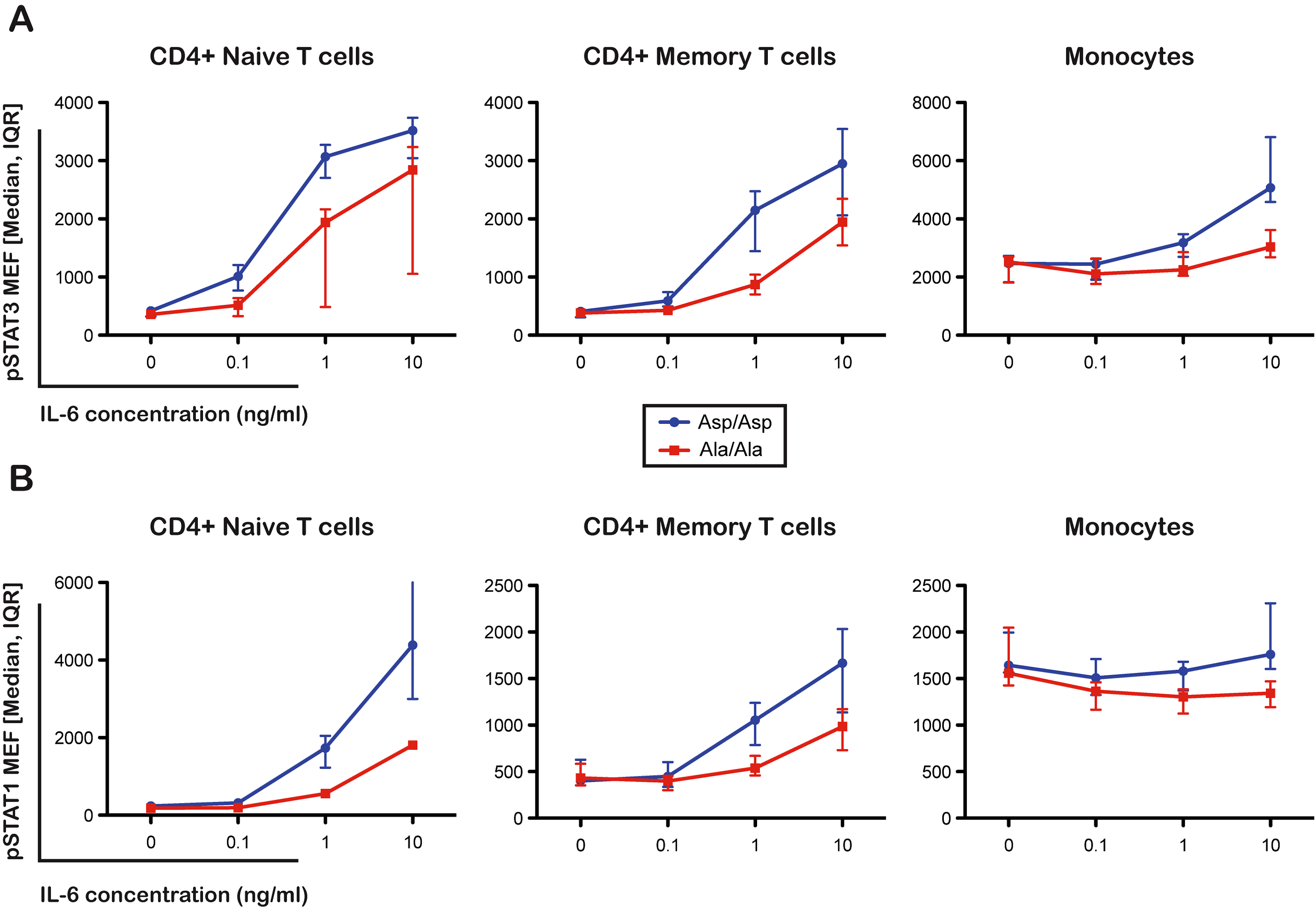

Supplement: Figure S7 — 358Ala is associated with decreased sensitivity to IL-6 signaling. Intracellular levels of pSTAT3 (A) and pSTAT1 (B) following stimulation of PBMCs with 0, 0.1, 1 or 10 ng/ml of IL-6. Activation of pSTAT3 and pSTAT1 was measured by flow cytometry in three distinct immune cell subsets, CD4+ naïve T cells, CD4+ memory T cells and monocytes in 14 358Asp and 14 358Ala homozygous volunteers from the Cambridge BioResource. Values represent the median and interquartile range (IQR) of the distribution of the fluorescence intensity values of pSTAT3/pSTAT1, measured as molecules of equivalent fluorochrome (MEF), in the two genotype groups for each dose of IL-6 stimulation. (TIF) [file pgen.1003444.s007.tif]

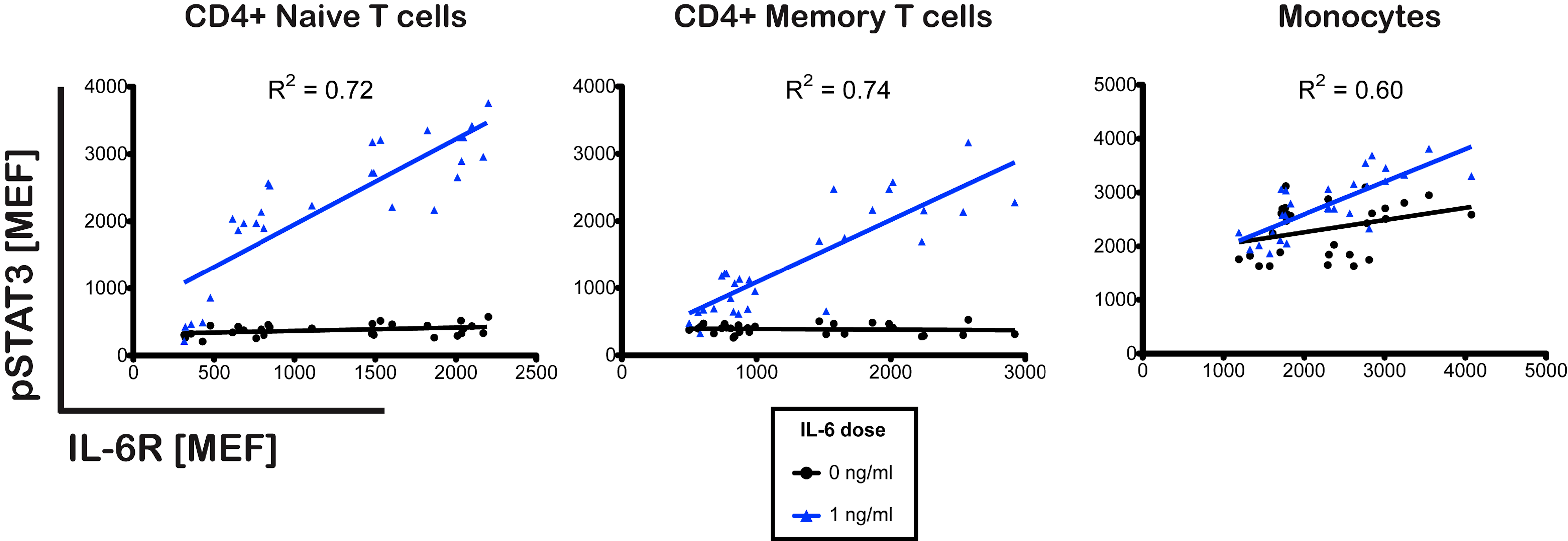

Supplement: Figure S8 — pSTAT3 activation is dependent on the surface levels of IL-6R. Correlation between surface levels of IL-6R and phosphorylated levels of intracellular STAT3 following stimulation with 0 ng/ml (depicted in black) or 1 ng/ml (depicted in blue) of IL-6, which was the dose where we found the strongest genotype-dependent effect of Asp358Ala on pSTAT3 activation. Intracellular levels of pSTAT3 were measured in CD4+ naïve and memory T cells and in monocytes from 14 Asp/Asp and 14 Ala/Ala homozygotes. R2, coefficient of determination; MEF, molecules of equivalent fluorochrome. (TIF) [file pgen.1003444.s008.tif]

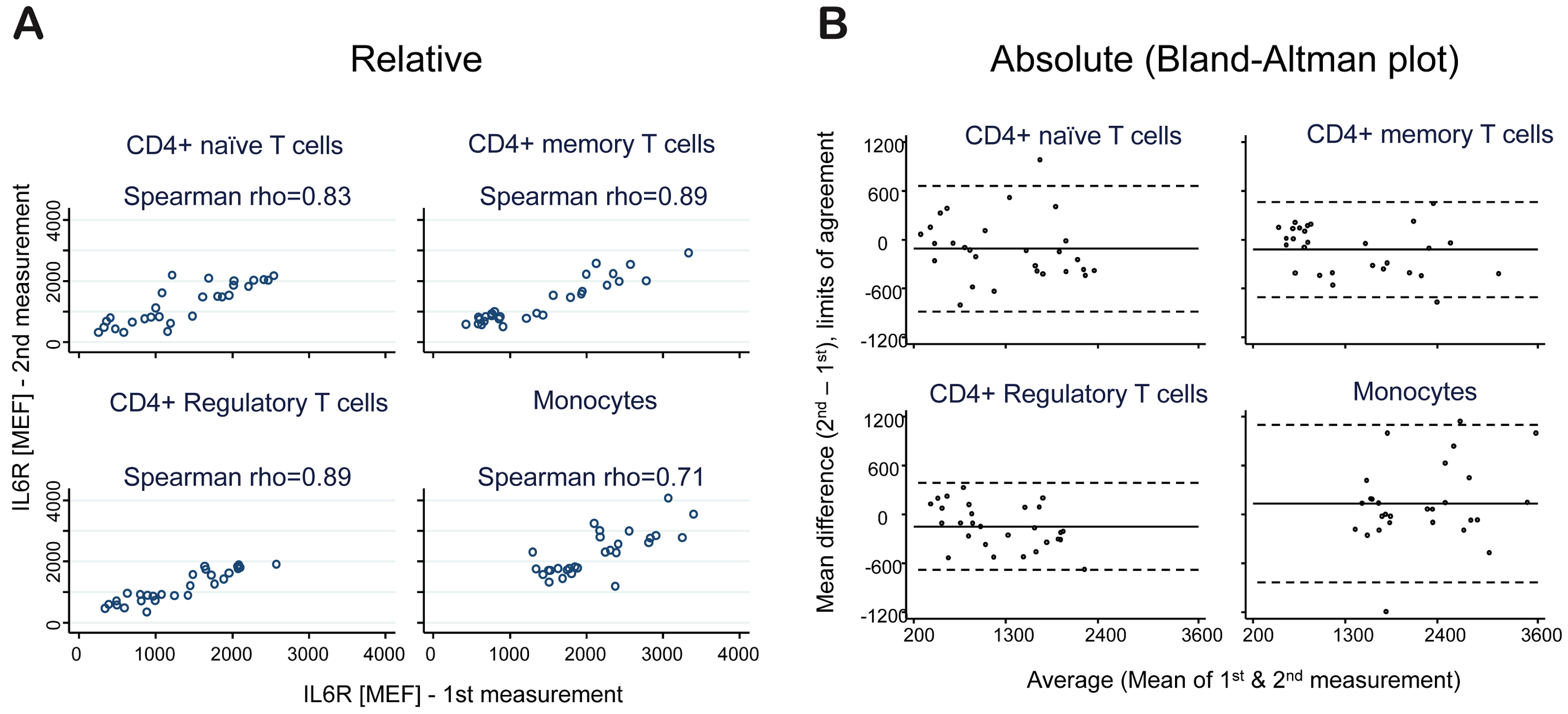

Supplement: Figure S9 — Repeatability of IL-6R measurement. In 28 donors, a second PBMC aliquot was measured again, approximately 1.5 months after the first measurement. (A) IL-6R expression from the first measurement (x-axis) versus the second measurement (y-axis). Correlation coefficients (Spearman's rho) of 0.71–0.89 indicate good correlation between the relative ordering of samples for two independent measurements. (B) Average of the two measurements, against the difference in measurements (2nd measurement – 1st measurement), together with the mean difference (solid line) and the limits of agreement (dashed lines) (Bland-Altman plot). On average the second measurement is lower than the first measurement, but there is no evidence for systematic bias. (TIF) [file pgen.1003444.s009.tif]

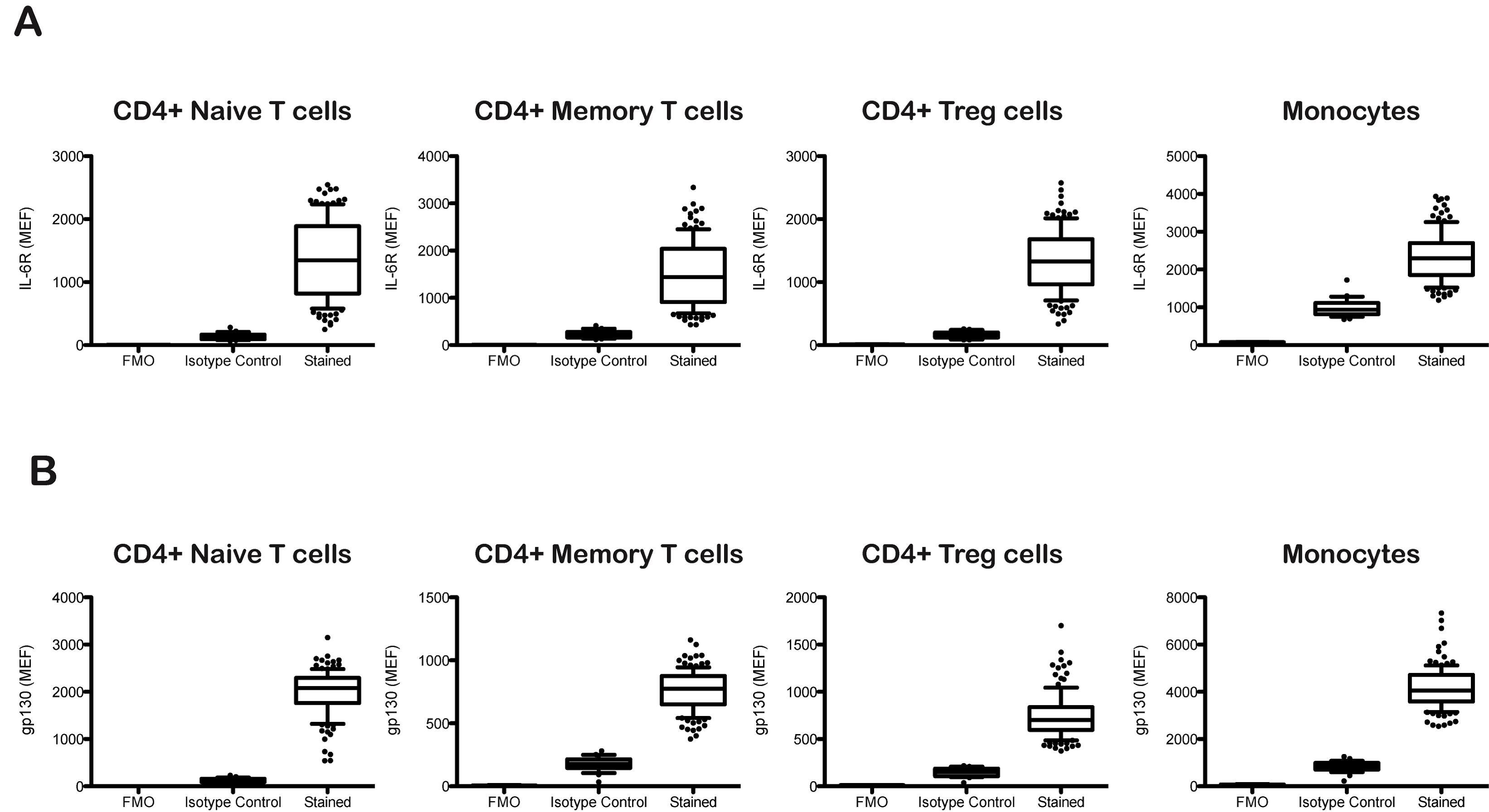

Supplement: Figure S10 — IL-6R and gp130 surface immunostainings distribution. Box and whisker plots depicting the distribution of the IL-6R (A) and gp130 (B) surface staining profiles on the unstained (FMO), isotype control and test sample groups. Error bars represent the 10–90 percentiles of the distribution. MEF, molecules of equivalent fluorochrome; FMO, fluorescence minus one. (TIF) [file pgen.1003444.s010.tif]
