# Supplementary material for: Functional IL6R 358Ala Allele Impairs Classical IL-6 Receptor Signaling and Influences Risk of Diverse Inflammatory Diseases
Source: PLoS Genet. 2013 Apr 4;9(4):e1003444. doi: 10.1371/journal.pgen.1003444 (PMC3617094; doi:10.1371/journal.pgen.1003444)
Supplement: Table S1 — Characteristics of the 4,249 individuals in whom circulating sIL-6R were measured. 3,605 samples were genotyped at 45 SNPs in the IL6R locus using the Illumina ImmunoChip. Mean (min-max) age-at-diagnosis of T1D cases (GRID) was 7.6 (0.6–16) years. Age = the age of the individual when the sample was taken. Note, there were plate effects (BR8000, P = 3.4×10^−4^) or box effects (GRID, P = 4.9×10^−77^; UKBS, P = 2.5×10^−5^; BR4000, P = 2.6×10^−13^), age effects (UKBS, P = 9.2×10^−5^; GRID, P = 0.0056) and for GRID, a dependence on the coefficient of variation (P = 7.6×10^−4^) on circulating sIL6R. GB, Great Britain; GRID, Genetic Resource Investigating Diabetes, UK BS, United Kingdom Blood Service. (DOCX) [file pgen.1003444.s011.docx]

**Table S1**: Characteristics of the 4,249 individuals in whom circulating sIL-6R were measured. 3,605 samples were genotyped at 45 SNPs in the *IL6R* locus using the Illumina ImmunoChip.

| Collection | Number of samples | Age  Mean (min-max) / years | Male  n (%) | Recruitment region | rs2228145 genotype  n (frequency) | | |
| --- | --- | --- | --- | --- | --- | --- | --- |
|  |  |  |  |  | Asp/Asp (A/A) | Asp/Ala (A/C) | Ala/Ala (C/C) |
| GRID | 1,512 | 14.7 (2-70) | 710 (47) | Across GB | 559 (0.38) | 682 (0.46) | 240 (0.16) |
| UK BS | 923 | 43 (17-69) | 493 (53) | Across GB | 291 (0.35) | 400 (0.48) | 137 (0.17) |
| BR4000 | 769 | 32.0 (17-49) | 291 (38) | East Anglia | 203 (0.36) | 285 (0.50) | 81 (0.14) |
| BR8000 | 1,045 | 38.8 (18-72) | 453 (43) | East Anglia | 256 (0.35) | 342 (0.47) | 129 (0.18) |

Mean (min-max) age-at-diagnosis of T1D cases (GRID) was 7.6 (0.6-16) years. Age = the age of the individual when the sample was taken. Note, there were plate effects (BR8000, *P* = 3.4x10^-4^) or box effects (GRID, *P* = 4.9x10^-77^; UKBS, *P* = 2.5x10^-5^; BR4000, *P* = 2.6x10^-13^), age effects (UKBS, *P* = 9.2x10^-5^; GRID, *P* = 0.0056) and for GRID, a dependence on the coefficient of variation (*P* = 7.6x10^-4^) on circulating sIL6R. GB, Great Britain; GRID, Genetic Resource Investigating Diabetes, UK BS, United Kingdom Blood Service.
