# Supplementary material for: Functional IL6R 358Ala Allele Impairs Classical IL-6 Receptor Signaling and Influences Risk of Diverse Inflammatory Diseases
Source: PLoS Genet. 2013 Apr 4;9(4):e1003444. doi: 10.1371/journal.pgen.1003444 (PMC3617094; doi:10.1371/journal.pgen.1003444)
Supplement: Table S2 — Association of sIL-6R with the IL6R region, in 3,605 samples. b = regression coefficient for a model containing all three SNPs. se = standard error of b. R2 (diff) = the difference in the square of the correlation coefficient for a model with and without the listed SNP. P is for the addition of the listed SNP to a model containing the other two SNPs. (DOCX) [file pgen.1003444.s012.docx]

**Table S2:** Association of sIL-6R with the *IL6R* region, in 3,605 samples.

| SNP | Genotype | N (frequency) | b (se) | % change (se) | R^2^(diff) | *P* |
| --- | --- | --- | --- | --- | --- | --- |
| rs2228145 | A/A | 1,309 (0.36) | 0 (reference) | Reference | 29.3% | <10^-300^ |
|  | A/C | 1,709 (0.47) | 0.129 (0.003) | +34.6 (0.7) |  |  |
|  | C/C | 587 (0.16) | 0.239 (0.004) | +73.4 (0.9) |  |  |
| rs4329505 | T/T | 2,517 (0.70) | 0 (reference) | Reference | 1.1% | 7.43x10^-29^ |
|  | T/C | 1,008 (0.28) | 0.031 (0.003) | +7.4 (0.7) |  |  |
|  | C/C | 80 (0.02) | 0.066 (0.009) | +16.4 (2.1) |  |  |
| rs1386821 | T/T | 2,340 (0.65) | 0 (reference) | Reference | 0.4% | 5.07x10^-11^ |
|  | T/G | 1,139 (0.32) | -0.016 (0.003) | -3.8 (0.7) |  |  |
|  | G/G | 126 (0.03) | -0.031 (0.007) | -7.4 (1.6) |  |  |

b = regression coefficient for a model containing all three SNPs. se=standard error of b. R^2^ (diff) = the difference in the square of the correlation coefficient for a model with and without the listed SNP. *P* is for the addition of the listed SNP to a model containing the other two SNPs.
