# Supplementary material for: Functional IL6R 358Ala Allele Impairs Classical IL-6 Receptor Signaling and Influences Risk of Diverse Inflammatory Diseases
Source: PLoS Genet. 2013 Apr 4;9(4):e1003444. doi: 10.1371/journal.pgen.1003444 (PMC3617094; doi:10.1371/journal.pgen.1003444)
Supplement: Table S3 — Characteristics of samples for qPCR expression analyses. P: Fisher's exact test. (DOCX) [file pgen.1003444.s013.docx]

**Table S3:** Characteristics of samples for qPCR expression analyses.

|  |  | ***IL6R* genotype (rs2228145)** | | | ***P*** |
| --- | --- | --- | --- | --- | --- |
|  |  | **Asp/Asp  (A/A)** | **Asp/Ala**  **(A/C)** | **Ala/Ala (C/C)** |  |
|  |  | ***n = 29*** | ***n = 44*** | ***n = 15*** |  |
| **Age band** |  |  |  |  | 0.2 |
|  | 20-29 | 5 | 3 | 0 |  |
|  | 30-39 | 10 | 13 | 6 |  |
|  | 40-49 | 9 | 18 | 9 |  |
|  | 50-59 | 5 | 10 | 0 |  |
| **Male (%)** |  | 10 (34.5) | 10 (22.7) | 5 (33.3) | 0.5 |

***P***: Fisher’s exact test.
