# Supplementary material for: Functional IL6R 358Ala Allele Impairs Classical IL-6 Receptor Signaling and Influences Risk of Diverse Inflammatory Diseases
Source: PLoS Genet. 2013 Apr 4;9(4):e1003444. doi: 10.1371/journal.pgen.1003444 (PMC3617094; doi:10.1371/journal.pgen.1003444)
Supplement: Table S4 — Association of IL6R SNPs with ds-IL6R expression, after accounting for rs2228145 in a subset (n = 75) of individuals with information on all three IL6R SNPs. Betas and P-values correspond to an additive model of inheritance (1 df test, 2 = effect allele) with rs2228145 plus one of the other SNPs (rs4329505 or rs1386821) in the model (columns: SNP, BetaSNP and P SNP). (DOCX) [file pgen.1003444.s014.docx]

**Table S4:** Association of *IL6R* SNPs with *ds-IL6R* expression, after accounting for rs2228145 in a subset (n = 75) of individuals with information on all three *IL6R* SNPs. Betas and *P*-values correspond to an additive model of inheritance (1 df test, 2 = effect allele) with rs2228145 plus one of the other SNPs (rs4329505 or rs1386821) in the model (columns: SNP, Beta_SNP_ and *P*_SNP_).

| **SNP** | **N Genotype** | | | **Beta _SNP_** | ***P* _SNP_** | **Beta _rs2228145_** | ***P*_rs2228145_** |
| --- | --- | --- | --- | --- | --- | --- | --- |
|  | ***1/1*** | ***1/2*** | ***2/2*** |  |  |  |  |
| rs2228145 | 28 | 35 | 12 | - | - | 0.034 | 1.22E-34 |
| rs4329505 | 2 | 19 | 54 | -0.0041 | 0.13 | 0.035 | 5.34E-35 |
| rs1386821 | 42 | 29 | 4 | -0.0024 | 0.39 | 0.034 | 2.31E-33 |
