# Supplementary material for: Functional IL6R 358Ala Allele Impairs Classical IL-6 Receptor Signaling and Influences Risk of Diverse Inflammatory Diseases
Source: PLoS Genet. 2013 Apr 4;9(4):e1003444. doi: 10.1371/journal.pgen.1003444 (PMC3617094; doi:10.1371/journal.pgen.1003444)
Supplement: Table S5 — Characteristics of samples for IL-6R surface expression. Samples have been selected and matched based on rs2228145 genotype (see methods for details). P-values reflect tests for differences across columns (Fisher's exact test). P: Fisher's exact test. (DOCX) [file pgen.1003444.s015.docx]

**Table S5:** Characteristics of samples for IL-6R surface expression. Samples have been selected and matched based on rs2228145 genotype (see Methods for details). *P*-values reflect tests for differences across columns (Fisher’s exact test).

|  |  | ***IL6R* genotype (rs2228145)** | | | ***P*** |
| --- | --- | --- | --- | --- | --- |
|  |  | **Asp/Asp  (A/A)** | **Asp/Ala**  **(A/C)** | **Ala/Ala (C/C)** |  |
|  |  | ***n = 64*** | ***n = 36*** | ***n = 28*** |  |
| **Age band** |  |  |  |  | 0.6 |
|  | 15-19 | 0 | 0 | 1 |  |
|  | 20-29 | 7 | 3 | 5 |  |
|  | 30-39 | 12 | 9 | 2 |  |
|  | 40-49 | 24 | 15 | 11 |  |
|  | 50-59 | 15 | 8 | 7 |  |
|  | 60+ | 6 | 1 | 2 |  |
| **Male (%)** |  | 19 (29.7) | 18 (50) | 9 (32.1) | 0.1 |
| **T1D (%)** |  | 15 (23.4) | 18 (50) | 3 (10.7) | 0.002 |

***P***: Fisher’s exact test
