# Supplementary material for: Functional IL6R 358Ala Allele Impairs Classical IL-6 Receptor Signaling and Influences Risk of Diverse Inflammatory Diseases
Source: PLoS Genet. 2013 Apr 4;9(4):e1003444. doi: 10.1371/journal.pgen.1003444 (PMC3617094; doi:10.1371/journal.pgen.1003444)
Supplement: Table S6 — Characteristics of samples for IL-6 stimulation. Samples have been selected and matched based on rs2228145 genotype (see methods for details). P: Fisher's exact test. (DOCX) [file pgen.1003444.s016.docx]

**Table S6:** Characteristics of samples for IL-6 stimulation. Samples have been selected and matched based on rs2228145 genotype (see Methods for details).

|  |  | **IL6R genotype (rs2228145)** | | ***P*** |
| --- | --- | --- | --- | --- |
|  |  | **Asp/Asp  (A/A)** | **Ala/Ala  (C/C)** |  |
|  |  | ***n = 14*** | ***n = 14*** |  |
| **Age band** |  |  |  | 1.0 |
|  | 20-29 | 0 | 1 |  |
|  | 30-39 | 1 | 1 |  |
|  | 40-49 | 7 | 6 |  |
|  | 50-59 | 5 | 5 |  |
|  | 60+ | 1 | 1 |  |
| **Male (%)** |  | 3 (21.4) | 3 (21.4%) | 1.0 |
| **T1D (%)** |  | 1 (7.1) | 1 (7.1) | 1.0 |

***P***: Fisher’s exact test
