# Supplementary material for: Functional IL6R 358Ala Allele Impairs Classical IL-6 Receptor Signaling and Influences Risk of Diverse Inflammatory Diseases
Source: PLoS Genet. 2013 Apr 4;9(4):e1003444. doi: 10.1371/journal.pgen.1003444 (PMC3617094; doi:10.1371/journal.pgen.1003444)
Supplement: Table S7 — Cell population frequencies of parent population according to rs2228145 genotype. Samples have been selected and matched based on rs2228145 genotype (see methods for details). * P-value derived from linear regression model, adjusting for sex, age (10-year bands), T1D status, and batch the sample was measured in. (DOCX) [file pgen.1003444.s017.docx]

**Table S7**: Cell population frequencies of parent population according to rs2228145 genotype. Samples have been selected and matched based on rs2228145 genotype (see Methods for details).

|  | **Asp/Asp (A/A)** | | **Asp/Ala (A/C)** | | **Ala/Ala (C/C)** | |  |
| --- | --- | --- | --- | --- | --- | --- | --- |
|  | *n = 64* | | *n = 36* | | *n =28* | |  |
| **Cell population / parent population (%)** | Mean | (SD) | Mean | (SD) | Mean | (SD) | *P*-value^*^ |
| **CD4+ cells / Lymphocytes** | 41.2 | (9.7) | 39.2 | (9.9) | 39.9 | (10.3) | 0.4 |
| **Memory T cells /  CD4+ cells** | 37.2 | (12.4) | 34.7 | (13.8) | 35.2 | (14.3) | 0.7 |
| **Naïve T cells /  CD4+ cells** | 40.0 | (13.4) | 41.9 | (16.8) | 41.9 | (14.9) | 0.9 |
| **Regulatory T cells / CD4+ cells** | 5.0 | (1.6) | 4.6 | (1.4) | 4.6 | (1.4) | 0.3 |

^*^*P*-value derived from linear regression model, adjusting for sex, age (10-year bands), T1D status, and batch the sample was measured in.
