# Supplementary material for: Functional IL6R 358Ala Allele Impairs Classical IL-6 Receptor Signaling and Influences Risk of Diverse Inflammatory Diseases
Source: PLoS Genet. 2013 Apr 4;9(4):e1003444. doi: 10.1371/journal.pgen.1003444 (PMC3617094; doi:10.1371/journal.pgen.1003444)
Supplement: Table S8 — Power calculations for the association of rs2228145 with T1D. Calculations were performed assuming a multiplicative model and an effect size (odds ratio) of 0.92, as estimated in our population of unrelated T1D patients (n = 8,371) and controls (n = 10,092). (DOCX) [file pgen.1003444.s018.docx]

**Table S8**: Power calculations for the association of rs2228145 with T1D. Calculations were performed assuming a multiplicative model and an effect size (odds ratio) of 0.92, as estimated in our population of unrelated T1D patients (n = 8,371) and controls (n = 10,092).

| **Significance level α** | **n cases** | **n controls** | **Power (%)** |
| --- | --- | --- | --- |
| 0.01 | 8,371 | 10,092 | >90 |
| 0.001 | 8,371 | 10.092 | 78 |
| 5.0 x 10^-8^ | 20,500 | 24,700 | 80 |
