# Supplementary material for: Functional IL6R 358Ala Allele Impairs Classical IL-6 Receptor Signaling and Influences Risk of Diverse Inflammatory Diseases
Source: PLoS Genet. 2013 Apr 4;9(4):e1003444. doi: 10.1371/journal.pgen.1003444 (PMC3617094; doi:10.1371/journal.pgen.1003444)
Supplement: Table S9 — Primers and probes sequences. (DOCX) [file pgen.1003444.s019.docx]

**Table S9:** Primers and probes sequences.

| *PCR Reaction* | *Primer/probe* | *Sequence* |
| --- | --- | --- |
| *β2 microglobulin (B2M)* | B2M-For | 5’-TGCTCGCGCTACTCTCTCT-3’ |
|  | B2M-Rev | 5’-TCCATTCTCTGCTGGATGAC-3’ |
|  | B2M-probe | 5’-CTGGAGGCTATCCAGCGTACTCCAA-3’ |
| *fl-Il6R* | fl-IL6R-For | 5’-TGCCATTGTTCTGAGGTTCAAG-3’ |
|  | fl-IL6R-Rev | 5’-ACCAGCTGCCCCAAAGAGT-3’ |
|  | fl-IL6R-probe | 5’-CAAGACAAGCATGCATCCGCCG-3’ |
| *ds-Il6R* | ds-IL6R-For | 5’-GACAAGCCTCCCAGGTTCAA-3’ |
|  | ds-IL6R-Rev | 5’-ACCAGCTGCCCCAAAGAGT-3’ |
|  | ds-IL6R-probe | 5’-CAAGACAAGCATGCATCCGCCG-3’ |
