# Supplementary material for: Functional IL6R 358Ala Allele Impairs Classical IL-6 Receptor Signaling and Influences Risk of Diverse Inflammatory Diseases
Source: PLoS Genet. 2013 Apr 4;9(4):e1003444. doi: 10.1371/journal.pgen.1003444 (PMC3617094; doi:10.1371/journal.pgen.1003444)
Supplement: Table S10 — Anti-human antibodies used for flow cytometry. (DOCX) [file pgen.1003444.s020.docx]

**Table S10:** Anti-human antibodies used for flow cytometry.

| *Panel* | *Antibody* | *Fluorochrome* | *Clone* | *Vendor* |
| --- | --- | --- | --- | --- |
| Surface Panel 1 | Anti-CD4 | AF-700 | RPA-T4 | BioLegend |
|  | Anti-CD127 | PE-Cy7 | eBioRDR5 | eBioscience |
|  | Anti-CD25 | BV 421 | BC96 | BioLegend |
|  | Anti-CD45RO | PerCP Cy5.5 | UCHL1 | BioLegend |
|  | Anti-CD14 | APC | M5E2 | BioLegend |
|  | Anti-HLA-DR | FITC | LN3 | BioLegend |
|  | Anti-CD126 | PE | UV4 | BioLegend |
| Surface Panel 2 | Anti-CD4 | AF-700 | RPA-T4 | BioLegend |
|  | Anti-CD127 | PE-Cy7 | eBioRDR5 | eBioscience |
|  | Anti-CD25 | FITC | 2A3 | BD Biosciences |
|  | Anti-CD25 | FITC | M-A251 | BD Biosciences |
|  | Anti-CD45RA | PB | HI100 | BioLegend |
|  | Anti-CD130 | PE | AM64 | BD Biosciences |
|  | Anti-CD126 | AF647 | BL-126 | BioLegend |
| Intracellular Panel 1 | Anti-CD4 | AF-700 | RPA-T4 | BioLegend |
|  | Anti-CD45RA | PB | HI100 | BioLegend |
|  | Anti-CD14 | APC | M5E2 | BioLegend |
|  | Anti-pSTAT3 (pY705) | AF488 | 4/P-STAT3 | BD Biosciences |
|  | Anti-pSTAT1 (pY701) | PE | 4a | BD Biosciences |
| Isotype Controls | Mouse IgG1k | PE | MOPC-21 | BD Biosciences |
|  | Mouse IgG1k | AF647 | MOPC-21 | BioLegend |
